# Supplementary material for: ERAP/HLA-C and KIR Genetic Profile in Couples with Recurrent Implantation Failure
Source: Int J Mol Sci. 2022 Oct 19;23(20):12518. doi: 10.3390/ijms232012518 (PMC9603896; doi:10.3390/ijms232012518)
Supplement: Supplementary file 1 [file ijms-23-12518-s001.zip › Supplementary Table S1.pdf]

Supplementary Table S1. Frequency of female *KIR* and male *HLA-C* gene combinations in couples undergoing *in vitro* fertilization and in fertile couples.

Female/male HLA-C	IVF	RIF	SIVF	Fertile
	N = 209 (%)	N = 120 (%)	N = 64 (%)	N = 146 (%)
AA/C1+	117 (55.98)	67 (55.83)	36 (56.25)	80 (54.79)
AA/C2+	92 (44.02)	53 (44.17)	28 (43.75)	66 (45.21)
	N = 138 (%)	N = 77 (%)	N = 44 (%)	N = 94 (%)
AA/C1C1	46 (33.33)	24 (31.17)	16 (36.36)	28 (29.79)
AA/C1C2	71 (51.45)	43 (55.84)	20 (45.45)	52 (55.32)
AA/C2C2	21 (15.22)	10 (12.99)	8 (18.18)	14 (14.89)
	N = 530 (%)	N = 293 (%)	N = 182 (%)	N = 336 (%)
Bx/C1+	302 (56.98)	171 (58.36)	98 (53.85)	192 (57.14)
Bx/C2+	228 (43.02)	122 (41.64)	84 (46.15)	144 (42.86)
	N = 353 (%)	N = 201 (%)	N = 117 (%)	N = 228 (%)
Bx/C1C1	125 (35.41)	**79 (39.30)**[a]	33 (28.21)	84 (36.84)
Bx/C1C2	177 (50.14)	92 (45.77)	65 (55.56)	108 (47.37)
Bx/C2C2	51 (14.45)	30 (14.93)	19 (16.24)	36 (15.79)
	N = 291 (%)	N = 167 (%)	N = 89 (%)	N = 220 (%)
CenAA/C1+	163 (56.01)	93 (55.69)	50 (56.18)	124 (56.36)
CenAA/C2+	128 (43.99)	74 (44.31)	39 (43.82)	96 (43.64)
	N = 195 (%)	N = 110 (%)	N = 61 (%)	N = 143 (%)
CenAA/C1C1	67 (34.36)	36 (32.73)	22 (36.07)	47 (32.87)
CenAA/C1C2	96 (49.23)	57 (51.82)	28 (45.90)	77 (53.85)
CenAA/C2C2	32 (16.41)	17 (15.45)	11 (18.03)	19 (13.29)
	N = 365 (%)	N = 202 (%)	N = 129 (%)	N = 196 (%)
CenAB/C1+	209 (57.26)	119 (58.91)	69 (53.49)	109 (55.61)
CenAB/C2+	156 (42.74)	83 (41.09)	60 (46.51)	87 (44.39)
	N = 241 (%)	N = 138 (%)	N = 82 (%)	N = 137 (%)
CenAB/C1C1	85 (35.27)	55 (39.86)	22 (26.83)	50 (36.50)
CenAB/C1C2	124 (51.45)	64 (46.38)	**47 (57.32)**[b]	59 (43.07)
CenAB/C2C2	32 (13.28)	19 (13.77)	13 (15.85)	28 (20.44)
	N = 83 (%)	N = 44 (%)	N = 28 (%)	N = 66 (%)
CenBB/C1+	47 (56.63)	26 (59.09)	15 (53.57)	39 (59.09)
CenBB/C2+	36 (43.37)	18 (40.91)	13 (46.43)	27 (40.91)
	N = 55 (%)	N = 30 (%)	N = 18 (%)	N = 42 (%)
CenBB/C1C1	19 (34.55)	12 (40.00)	5 (27.78)	15 (35.71)
CenBB/C1C2	28 (50.91)	14 (46.67)	10 (55.56)	24 (57.14)
CenBB/C2C2	8 (14.55)	4 (13.33)	3 (16.67)	3 (7.14)
	N = 436 (%)	N = 255 (%)	N = 133 (%)	N = 266 (%)
TelAA/C1+	247 (56.65)	146 (57.25)	73 (54.89)	144 (54.14)
TelAA/C2+	189 (43.35)	109 (42.75)	60 (45.11)	122 (45.86)
	N = 285 (%)	N = 163 (%)	N = 90 (%)	N = 175 (%)
TelAA/C1C1	96 (33.68)	54 (33.13)	30 (33.33)	53 (30.29)
TelAA/C1C2	151 (52.98)	92 (56.44)	43 (47.78)	91 (52.00)
TelAA/C2C2	38 (13.33)	17 (10.43)	17 (18.89)	31 (17.71)
	N = 253 (%)	N = 138 (%)	N = 91 (%)	N = 185 (%)

Female/male HLA-C	IVF	RIF	SIVF	Fertile
TelAB/C1+	143 (56.52)	78 (56.52)	50 (54.95)	111 (60.00)
TelAB/C2+	110 (43.48)	60 (43.48)	41 (45.05)	74 (40.00)
	N = 173 (%)	N = 99 (%)	N = 58 (%)	N = 129 (%)
TelAB/C1C1	63 (36.42)	39 (39.39)	17 (29.31)	55 (42.64)
TelAB/C1C2	80 (46.24)	**39 (39.39)**[c]	33 (56.90)	56 (43.41)
TelAB/C2C2	30 (17.34)	21 (21.21)	8 (13.79)	18 (13.95)
	N = 50 (%)	N = 20 (%)	N = 22 (%)	N = 29 (%)
TelBB/C1+	29 (58.00)	14 (70.00)	11 (50.00)	16 (55.17)
TelBB/C2+	21 (42.00)	6 (30.00)	11 (50.00)	13 (44.83)
	N = 33 (%)	N = 16 (%)	N = 13 (%)	N = 17 (%)
TelBB/C1C1	12 (36.36)	**10 (62.50)**[d.e]	2 (15.38)	4 (23.53)
TelBB/C1C2	17 (51.52)	**4 (25.00)**[f.g]	9 (69.23)	12 (70.59)
TelBB/C2C2	4 (12.12)	2 (12.50)	2 (15.38)	1 (5.88)
	N = 207 (%)	N = 120 (%)	N = 62 (%)	N = 146 (%)
CenAA/TelAA/C1+	116 (56.04)	67 (55.83)	35 (56.45)	80 (54.79)
CenAA/TelAA/C2+	91 (43.96)	53 (44.17)	27 (43.55)	66 (45.21)
	N = 137 (%)	N = 77 (%)	N = 43 (%)	N = 94 (%)
CenAA/TelAA/C1C1	46 (33.58)	24 (31.17)	16 (37.21)	28 (29.79)
CenAA/TelAA/C1C2	70 (51.09)	43 (55.84)	19 (44.19)	52 (55.32)
CenAA/TelAA/C2C2	21 (15.33)	10 (12.99)	8 (18.60)	14 (14.89)
	N = 76 (%)	N = 44 (%)	N = 22 (%)	N = 66 (%)
CenAA/TelAB/C1+	43 (56.58)	24 (54.55)	13 (59.09)	40 (60.61)
CenAA/TelAB/C2+	33 (43.42)	20 (45.45)	9 (40.91)	26 (39.39)
	N = 53 (%)	N = 31 (%)	N = 15 (%)	N = 45 (%)
CenAA/TelAB/C1C1	20 (37.74)	11 (35.48)	6 (40.00)	19 (42.22)
CenAA/TelAB/C1C2	23 (43.40)	13 (41.94)	7 (46.67)	21 (46.67)
CenAA/TelAB/C2C2	10 (18.87)	7 (22.58)	2 (13.33)	5 (11.11)
	N = 8 (%)	N = 3 (%)	N = 5 (%)	N = 6 (%)
CenAA/TelBB/C1+	4 (50.00)	2 (66.67)	2 (40.00)	3 (50.00)
CenAA/TelBB/C2+	4 (50.00)	1 (33.33)	3 (60.00)	3 (50.00)
	N = 5 (%)	N = 2 (%)	N = 3 (%)	N = 3 (%)
CenAA/TelBB/C1C1	1 (20.00)	1 (50.00)	0 (0.00)	0 (0.00)
CenAA/TelBB/C1C2	3 (60.00)	1 (50.00)	2 (66.67)	3 (100.00)
CenAA/TelBB/C2C2	1 (20.00)	0 (0.00)	1 (33.33)	0 (0.00)
	N = 193 (%)	N = 112 (%)	N = 65 (%)	N = 98 (%)
CenAB/TelAA/C1+	110 (56.99)	64 (57.14)	35 (53.85)	50 (51.02)
CenAB/TelAA/C2+	83 (43.01)	48 (42.86)	30 (46.15)	48 (48.98)
	N = 124 (%)	N = 71 (%)	N = 42 (%)	N = 66 (%)
CenAB/TelAA/C1C1	41 (33.06)	23 (32.39)	12 (28.57)	18 (27.27)
CenAB/TelAA/C1C2	69 (55.65)	41 (57.75)	23 (54.76)	32 (48.48)
CenAB/TelAA/C2C2	**14 (11.29)**[h]	**7 (9.86)**[i]	7 (16.67)	16 (24.24)
	N = 145 (%)	N = 78 (%)	N = 57 (%)	N = 87 (%)
CenAB/TelAB/C1+	84 (57.93)	47 (60.26)	31 (54.39)	52 (59.77)
CenAB/TelAB/C2+	61 (42.07)	31 (39.74)	26 (45.61)	35 (40.23)
	N = 100 (%)	N = 58 (%)	N = 36 (%)	N = 64 (%)
CenAB/TelAB/C1C1	39 (39.00)	27 (46.55)	10 (27.78)	29 (45.31)
CenAB/TelAB/C1C2	45 (45.00)	**20 (34.48)**[j]	**21 (58.33)**[k]	23 (35.94)

Female/male HLA-C	IVF	RIF	SIVF	Fertile
CenAB/TelAB/C2C2	16 (16.00)	11 (18.97)	5 (13.89)	12 (18.75)
	N = 27 (%)	N = 12 (%)	N = 7 (%)	N = 11 (%)
CenAB/TelBB/C1+	15 (55.56)	8 (66.67)	3 (42.86)	7 (63.64)
CenAB/TelBB/C2+	12 (44.44)	4 (33.33)	4 (57.14)	4 (36.36)
	N = 17 (%)	N = 9 (%)	N = 4 (%)	N = 7 (%)
CenAB/TelBB/C1C1	5 (29.41)	5 (55.56)	0 (0.00)	3 (42.86)
CenAB/TelBB/C1C2	10 (58.82)	3 (33.33)	3 (75.00)	4 (57.14)
CenAB/TelBB/C2C2	2 (11.76)	1 (11.11)	1 (25.00)	0 (0.00)
	N = 36 (%)	N = 23 (%)	N = 6 (%)	N = 22 (%)
CenBB/TelAA/C1+	21 (58.33)	15 (65.22)	3 (50.00)	14 (63.64)
CenBB/TelAA/C2+	15 (41.67)	8 (34.78)	3 (50.00)	8 (36.36)
	N = 24 (%)	N = 15 (%)	N = 5 (%)	N = 15 (%)
CenBB/TelAA/C1C1	9 (37.50)	7 (46.67)	2 (40.00)	7 (46.67)
CenBB/TelAA/C1C2	12 (50.00)	8 (53.33)	1 (20.00)	7 (46.67)
CenBB/TelAA/C2C2	3 (12.50)	0 (0.00)[l]	2 (40.00)	1 (6.67)
	N = 32 (%)	N = 16 (%)	N = 12 (%)	N = 32 (%)
CenBB/TelAB/C1+	16 (50.00)	7 (43.75)	6 (50.00)	19 (59.38)
CenBB/TelAB/C2+	16 (50.00)	9 (56.25)	6 (50.00)	13 (40.62)
	N = 20 (%)	N = 10 (%)	N = 7 (%)	N = 20 (%)
CenBB/TelAB/C1C1	4 (20.00)	1 (10.00)	1 (14.29)	7 (35.00)
CenBB/TelAB/C1C2	12 (60.00)	6 (60.00)	5 (71.43)	12 (60.00)
CenBB/TelAB/C2C2	4 (20.00)	3 (30.00)	1 (14.29)	1 (5.00)
	N = 15 (%)	N = 5 (%)	N = 10 (%)	N = 12 (%)
CenBB/TelBB/C1+	10 (66.67)	4 (80.00)	6 (60.00)	6 (50.00)
CenBB/TelBB/C2+	5 (33.33)	1 (20.00)	4 (40.00)	6 (50.00)
	N = 11 (%)	N = 5 (%)	N = 6 (%)	N = 7 (%)
CenBB/TelBB/C1C1	6 (54.55)	4 (80.00)	2 (33.33)	1 (14.29)
CenBB/TelBB/C1C2	4 (36.36)	0 (0.00)[m]	4 (66.67)	5 (71.43)
CenBB/TelBB/C2C2	1 (9.09)	1 (20.00)	0 (0.00)	1 (14.29)

IVF-ET – in vitro fertilization embryo transfer; RIF – recurrent implantation failure; SIVF – successful pregnancy after IVF-ET; p – probability; $p_{corr.}$ – probability after Bonferroni correction for multiple comparisons (x3 for possible combinations with HLA-C); OR – odds ratio; 95% CI – confidence interval from two-sided Fisher's exact test; ns – not significant. Values in bold indicate significant differences.

RIF *vs.* SIVF: [a]$p/p_{corr.}$ = 0.052/ns, OR = 1.646, 95% CI (0.98-2.79); [c]$p/p_{corr.}$ = 0.046/ns, OR = 0.495, 95% CI (0.24-1.00); [e]$p/p_{corr.}$ = 0.022/ns, OR = 8.412, 95% CI (1.21-104.09); [g]$p/p_{corr.}$ = 0.027/ns, OR = 0.160, 95% CI (0.02-0.96); [j]$p/p_{corr.}$ = 0.032/ns, OR = 0.38, 95% CI (0.15-0.96); [l]$p/p_{corr.}$ = 0.053/ns, OR = 0.000, 95% CI (0.00-1.60)

SIVF *vs.* Fertile: [b]$p/p_{corr.}$ = 0.051/ns, OR = 1.771, 95% CI (0.98-3.21); [k]$p/p_{corr.}$ = 0.037/ns, OR = 2.472, 95% CI (1.00-6.28);

RIF *vs.* Fertile: [d]$p/p_{corr.}$ = 0.037/ns, OR = 5.115, 95% CI (0.98-32.72); [f]$p/p_{corr.}$ = 0.015/ns, OR = 0.149, 95% CI (0.02-0.80); [i]$p/p_{corr.}$ = 0.038/ns, OR = 0.344, 95% CI (0.11-0.97); [m]$p/p_{corr.}$ = 0.028/ns, OR = 0.000, 95% CI (0.00-1.03);

IVF *vs.* Fertile: [h]$p/p_{corr.}$ = 0.035/ns, OR = 0.400, 95% CI (0.17-0.95).
