# Supplementary material for: ERAP/HLA-C and KIR Genetic Profile in Couples with Recurrent Implantation Failure
Source: Int J Mol Sci. 2022 Oct 19;23(20):12518. doi: 10.3390/ijms232012518 (PMC9603896; doi:10.3390/ijms232012518)
Supplement: Supplementary file 1 [file ijms-23-12518-s001.zip › Supplementary Table S2.pdf]

Supplementary Table S2. Distribution of female *ERAP* and male *HLA-C* genotype combination in couples undergoing *in vitro* fertilization and in fertile couples.

Female ERAP/male HLA-C	IVF	RIF	SIVF	Fertile
ERAP1 rs30187/HLA-C	N = 419 (%)	N = 238 (%)	N = 134 (%)	N = 272 (%)
CC/C1+	181 (43.20)	96 (40.34)	59 (44.03)	128 (47.06)
CT/C1+	188 (44.87)	111 (46.64)	59 (44.03)	122 (44.85)
TT/C1+	50 (11.93)	31 (13.03)	16 (11.94)	22 (8.09)
	N = 320 (%)	N = 175 (%)	N = 112 (%)	N = 210 (%)
CC/C2+	141 (44.06)	77 (44.00)	45 (40.18)	106 (50.48)
CT/C2+	137 (42.81)	74 (42.29)	53 (47.32)	89 (42.38)
TT/C2+	**42 (13.12)**[a]	**24 (13.71)**[b]	14 (12.50)	15 (7.14)
	N = 171 (%)	N = 103 (%)	N = 49 (%)	N = 112 (%)
CC/C1C1	79 (46.20)	**40 (38.83)**[c]	**30 (61.22)**[d]	46 (41.07)
CT/C1C1	78 (45.61)	52 (50.49)	17 (34.69)	53 (47.32)
TT/C1C1	14 (8.19)	11 (10.68)	2 (4.08)	13 (11.61)
	N = 248 (%)	N = 135 (%)	N = 85 (%)	N = 160 (%)
CC/C1C2	**102 (41.13)**[e]	56 (41.48)	**29 (34.12)**[f]	82 (51.25)
CT/C1C2	110 (44.35)	59 (43.70)	42 (49.41)	69 (43.12)
TT/C1C2	**36 (14.52)**[g]	**20 (14.81)**[h]	**14 (16.47)**[i]	9 (5.62)
	N = 72 (%)	N = 40 (%)	N = 27 (%)	N = 50 (%)
CC/C2C2	39 (54.17)	21 (52.50)	16 (59.26)	24 (48.00)
CT/C2C2	27 (37.50)	15 (37.50)	11 (40.74)	20 (40.00)
TT/C2C2	6 (8.33)	4 (10.00)	0 (0.00)	6 (12.00)
ERAP1 rs27044/HLA-C	N = 419 (%)	N = 238 (%)	N = 134 (%)	N = 272 (%)
CC/C1+	221 (52.74)	**117 (49.16)**[j]	72 (53.73)	159 (58.46)
CG/C1+	166 (39.62)	101 (42.44)	52 (38.81)	95 (34.93)
GG/C1+	32 (7.64)	20 (8.40)	10 (7.46)	18 (6.62)
	N = 320 (%)	N = 175 (%)	N = 112 (%)	N = 210 (%)
CC/C2+	**164 (51.25)**[k]	**89 (50.86)**[l]	**52 (46.43)**[m]	130 (61.90)
CG/C2+	**129 (40.31)**[n]	71 (40.57)	**51 (45.54)**[o]	66 (31.43)
GG/C2+	27 (8.44)	15 (8.57)	9 (8.04)	14 (6.67)
	N = 171 (%)	N = 103 (%)	N = 49 (%)	N = 112 (%)
CC/C1C1	98 (57.31)	**50 (48.54)**[p]	**36 (73.47)**[q]	59 (52.68)
CG/C1C1	65 (38.01)	**46 (44.66)**[r]	12 (24.49)	44 (39.29)
GG/C1C1	8 (4.68)	7 (6.80)	1 (2.04)	9 (8.04)
	N = 248 (%)	N = 135 (%)	N = 85 (%)	N = 160 (%)
CC/C1C2	**123 (49.60)**[s]	**67 (49.63)**[t]	**36 (42.35)**[u]	100 (62.50)
CG/C1C2	101 (40.73)	55 (40.74)	**40 (47.06)**[w]	51 (31.88)
GG/C1C2	24 (9.68)	13 (9.63)	9 (10.59)	9 (5.62)
	N = 72 (%)	N = 40 (%)	N = 27 (%)	N = 50 (%)
CC/C2C2	41 (56.94)	22 (55.00)	16 (59.26)	30 (60.00)
CG/C2C2	28 (38.89)	16 (40.00)	11 (40.74)	15 (30.00)
GG/C2C2	3 (4.17)	2 (5.00)	0 (0.00)	5 (10.00)
ERAP1 rs26653/HLA-C	N = 419 (%)	N = 238 (%)	N = 134 (%)	N = 269 (%)
GG/C1+	230 (54.89)	128 (53.78)	73 (54.48)	148 (55.02)
CG/C1+	169 (40.33)	99 (41.60)	56 (41.79)	106 (39.41)

Female ERAP/male HLA-C	IVF	RIF	SIVF	Fertile
CC/C1+	20 (4.77)	11 (4.62)	5 (3.73)	15 (5.58)
	N = 320 (%)	N = 175 (%)	N = 112 (%)	N = 208 (%)
GG/C2+	175 (54.69)	95 (54.29)	59 (52.68)	121 (58.17)
CG/C2+	128 (40.00)	71 (40.57)	49 (43.75)	78 (37.50)
CC/C2+	17 (5.31)	9 (5.14)	4 (3.57)	9 (4.33)
	N = 171 (%)	N = 103 (%)	N = 49 (%)	N = 111 (%)
GG/C1C1	92 (53.80)	51 (49.51)	30 (61.22)	58 (52.25)
CG/C1C1	71 (41.52)	47 (45.63)	17 (34.69)	45 (40.54)
CC/C1C1	8 (4.68)	5 (4.85)	2 (4.08)	8 (7.21)
	N = 248 (%)	N = 135 (%)	N = 85 (%)	N = 158 (%)
GG/C1C2	138 (55.65)	77 (57.04)	43 (50.59)	90 (56.96)
CG/C1C2	98 (39.52)	52 (38.52)	39 (45.88)	61 (38.61)
CC/C1C2	12 (4.84)	6 (4.44)	3 (3.53)	7 (4.43)
	N = 72 (%)	N = 40 (%)	N = 27 (%)	N = 50 (%)
GG/C2C2	37 (51.39)	18 (45.00)	16 (59.26)	31 (62.00)
CG/C2C2	30 (41.67)	19 (47.50)	10 (37.04)	17 (34.00)
CC/C2C2	5 (6.94)	3 (7.50)	1 (3.70)	2 (4.00)
ERAP1 rs26618/HLA-C	N = 419 (%)	N = 238 (%)	N = 134 (%)	N = 272 (%)
TT/C1+	221 (52.74)	132 (55.46)	68 (50.75)	129 (47.43)
CT/C1+	166 (39.62)	89 (37.39)	55 (41.04)	120 (44.12)
CC/C1+	32 (7.64)	17 (7.14)	11 (8.21)	23 (8.46)
	N = 320 (%)	N = 175 (%)	N = 112 (%)	N = 210 (%)
TT/C2+	171 (53.44)	97 (55.43)	60 (53.57)	101 (48.10)
CT/C2+	124 (38.75)	66 (37.71)	42 (37.50)	90 (42.86)
CC/C2+	25 (7.81)	12 (6.86)	10 (8.93)	19 (9.05)
	N = 171 (%)	N = 103 (%)	N = 49 (%)	N = 112 (%)
TT/C1C1	93 (54.39)	62 (60.19)	21 (42.86)	54 (48.21)
CT/C1C1	64 (37.43)	33 (32.04)	23 (46.94)	50 (44.64)
CC/C1C1	14 (8.19)	8 (7.77)	5 (10.20)	8 (7.14)
	N = 248 (%)	N = 135 (%)	N = 85 (%)	N = 160 (%)
TT/C1C2	128 (51.61)	70 (51.85)	47 (55.29)	75 (46.88)
CT/C1C2	102 (41.13)	56 (41.48)	32 (37.65)	70 (43.75)
CC/C1C2	18 (7.26)	9 (6.67)	6 (7.06)	15 (9.38)
	N = 72 (%)	N = 40 (%)	N = 27 (%)	N = 50 (%)
TT/C2C2	43 (59.72)	27 (67.50)	13 (48.15)	26 (52.00)
CT/C2C2	22 (30.56)	10 (25.00)	10 (37.04)	20 (40.00)
CC/C2C2	7 (9.72)	3 (7.50)	4 (14.81)	4 (8.00)
ERAP1 rs2287987/HLA-C	N = 419 (%)	N = 238 (%)	N = 134 (%)	N = 272 (%)
TT/C1+	258 (61.58)	146 (61.34)	87 (64.93)	169 (62.13)
CT/C1+	142 (33.89)	81 (34.03)	41 (30.60)	96 (35.29)
CC/C1+	19 (4.53)	11 (4.62)	6 (4.48)	7 (2.57)
	N = 320 (%)	N = 175 (%)	N = 112 (%)	N = 210 (%)
TT/C2+	205 (64.06)	111 (63.43)	74 (66.07)	122 (58.10)
CT/C2+	98 (30.62)^{x}	55 (31.43)	32 (28.57)	82 (39.05)
CC/C2+	17 (5.31)	9 (5.14)	6 (5.36)	6 (2.86)
	N = 171 (%)	N = 103 (%)	N = 49 (%)	N = 112 (%)
TT/C1C1	99 (57.89)	61 (59.22)	29 (59.18)	76 (67.86)

Female ERAP/male HLA-C	IVF	RIF	SIVF	Fertile
CT/C1C1	65 (38.01)	37 (35.92)	18 (36.73)	34 (30.36)
CC/C1C1	7 (4.09)	5 (4.85)	2 (4.08)	2 (1.79)
	N = 248 (%)	N = 135 (%)	N = 85 (%)	N = 160 (%)
TT/C1C2	159 (64.11)	85 (62.96)	58 (68.24)	93 (58.12)
CT/C1C2	77 (31.05)	44 (32.59)	23 (27.06)	62 (38.75)
CC/C1C2	12 (4.84)	6 (4.44)	4 (4.71)	5 (3.12)
	N = 72 (%)	N = 40 (%)	N = 27 (%)	N = 50 (%)
TT/C2C2	46 (63.89)	26 (65.00)	16 (59.26)	29 (58.00)
CT/C2C2	21 (29.17)	11 (27.50)	9 (33.33)	20 (40.00)
CC/C2C2	5 (6.94)	3 (7.50)	2 (7.41)	1 (2.00)
ERAP2 rs2248374/HLA-C	N = 418 (%)	N = 237 (%)	N = 134 (%)	N = 269 (%)
AA/C1+	108 (25.84)	58 (24.47)	40 (29.85)	72 (26.77)
AG/C1+	204 (48.80)	114 (48.10)	64 (47.76)	133 (49.44)
GG/C1+	106 (25.36)	65 (27.43)	30 (22.39)	64 (23.79)
	N = 319 (%)	N = 174 (%)	N = 112 (%)	N = 208 (%)
AA/C2+	79 (24.76)	41 (23.56)	33 (29.46)	54 (25.96)
AG/C2+	150 (47.02)	81 (46.55)	50 (44.64)	100 (48.08)
GG/C2+	90 (28.21)	52 (29.89)	29 (25.89)	54 (25.96)
	N = 171 (%)	N = 103 (%)	N = 49 (%)	N = 111 (%)
AA/C1C1	47 (27.49)	26 (25.24)	15 (30.61)	32 (28.83)
AG/C1C1	85 (49.71)	51 (49.51)	26 (53.06)	54 (48.65)
GG/C1C1	39 (22.81)	26 (25.24)	8 (16.33)	25 (22.52)
	N = 247 (%)	N = 134 (%)	N = 85 (%)	N = 158 (%)
AA/C1C2	61 (24.70)	32 (23.88)	25 (29.41)	40 (25.32)
AG/C1C2	119 (48.18)	63 (47.01)	38 (44.71)	79 (50.00)
GG/C1C2	67 (27.13)	39 (29.10)	22 (25.88)	39 (24.68)
	N = 72 (%)	N = 40 (%)	N = 27 (%)	N = 50 (%)
AA/C2C2	18 (25.00)	9 (22.50)	8 (29.63)	14 (28.00)
AG/C2C2	31 (43.06)	18 (45.00)	12 (44.44)	21 (42.00)
GG/C2C2	23 (31.94)	13 (32.50)	7 (25.93)	15 (30.00)
ERAP1 rs6861666/HLA-C	N = 418 (%)	N = 237 (%)	N = 134 (%)	N = 267 (%)
AA/C1+	361 (86.36)	202 (85.23)	115 (85.82)	229 (85.77)
AG/C1+	55 (13.16)	34 (14.35)	18 (13.43)	38 (14.23)
GG/C1+	2 (0.48)	1 (0.42)	1 (0.75)	0 (0.00)
	N = 319 (%)	N = 174 (%)	N = 112 (%)	N = 207 (%)
AA/C2+	272 (85.27)	149 (85.63)	94 (83.93)	174 (84.06)
AG/C2+	46 (14.42)	25 (14.37)	17 (15.18)	32 (15.46)
GG/C2+	1 (0.31)	0 (0.00)	1 (0.89)	1 (0.48)
	N = 171 (%)	N = 103 (%)	N = 49 (%)	N = 110 (%)
AA/C1C1	148 (86.55)	85 (82.52)	44 (89.80)	94 (85.45)
AG/C1C1	22 (12.87)	17 (16.50)	5 (10.20)	16 (14.55)
GG/C1C1	1 (0.58)	1 (0.97)	0 (0.00)	0 (0.00)
	N = 247 (%)	N = 134 (%)	N = 85 (%)	N = 157 (%)
AA/C1C2	213 (86.23)	117 (87.31)	71 (83.53)	135 (85.99)
AG/C1C2	33 (13.36)	17 (12.69)	13 (15.29)	22 (14.01)
GG/C1C2	1 (0.40)	0 (0.00)	1 (1.18)	0 (0.00)
	N = 72 (%)	N = 40 (%)	N = 27 (%)	N = 50 (%)

Female ERAP/male HLA-C	IVF	RIF	SIVF	Fertile
AA/C2C2	59 (81.94)	32 (80.00)	23 (85.19)	39 (78.00)
AG/C2C2	13 (18.06)	8 (20.00)	4 (14.81)	10 (20.00)
GG/C2C2	0 (0.00)	0 (0.00)	0 (0.00)	1 (2.00)

IVF-ET – in vitro fertilization embryo transfer; RIF – recurrent implantation failure; SIVF – successful pregnancy after IVF-ET; p – probability; $p_{corr.}$ – probability after Bonferroni correction for multiple comparisons (x6 for possible combinations with HLA-C alleles or x9 for possible combinations with HLA-C genotypes); OR – odds ratio; 95% CI – confidence interval from two-sided Fisher's exact test; ns – not significant. Values in bold indicate significant differences.

IVF *vs.* Fertile: $^{a}p/p_{corr.}$ = 0.032/ns, OR = 1.962, 95% CI (1.03-3.92); $^{e}p/p_{corr.}$ = 0.053/ns, OR = 0.665, 95% CI (0.44-1.01); $^{g}p/p_{corr.}$ = 0.006/0.050, OR = 2.843, 95% CI (1.3-6.92); $^{k}p/p_{corr.}$ = 0.016/ns, OR = 0.647, 95% CI (0.45-0.94); $^{n}p/p_{corr.}$ = 0.043/ns, OR = 1.472, 95% CI (1.01-2.17); $^{s}p/p_{corr.}$ = 0.011/ns, OR = 0.591, 95% CI (0.39-0.9); $^{x}p/p_{corr.}$ = 0.049/ns, OR = 0.690, 95% CI (0.47-1.01);

RIF *vs.* Fertile: $^{b}p/p_{corr.}$ = 0.041/ns, OR = 2.062, 95% CI (1-4.39); $^{h}p/p_{corr.}$ = 0.010/ns, OR = 2.907, 95% CI (1.21-7.54); $^{j}p/p_{corr.}$ = 0.041/ns, OR = 0.688, 95% CI (0.48-0.99); $^{l}p/p_{corr.}$ = 0.031/ns, OR = 0.638, 95% CI (0.42-0.98); $^{t}p/p_{corr.}$ = 0.034/ns, OR = 0.592, 95% CI (0.36-0.97);

RIF *vs.* SIVF: $^{c}p/p_{corr.}$ = 0.014/ns, OR = 0.405, 95% CI (0.19-0.85); $^{p}p/p_{corr.}$ = 0.005/0.044, OR = 0.343, 95% CI (0.15-0.75); $^{r}p/p_{corr.}$ = 0.020/ns, OR = 2.474, 95% CI (1.11-5.83);

SIVF *vs.* Fertile: $^{d}p/p_{corr.}$ = 0.025/ns, OR = 2.254, 95% CI (1.08-4.79); $^{f}p/p_{corr.}$ = 0.011/ns, OR = 0.494, 95% CI (0.27-0.88); $^{i}p/p_{corr.}$ = 0.010/ns, OR = 3.290. 95% CI (1.26-9.06); $^{m}p/p_{corr.}$ = 0.009/ns, OR = 0.534, 95% CI (0.33-0.87); $^{o}p/p_{corr.}$ = 0.015/ns, OR = 1.821, 95% CI (1.1-3); $^{q}p/p_{corr.}$ = 0.015/ns, OR = 2.474, 95% CI (1.14-5.65); $^{u}p/p_{corr.}$ = 0.003/0.027, OR = 0.442, 95% CI (0.25-0.78); $^{w}p/p_{corr.}$ = 0.026/ns, OR = 1.895, 95% CI (1.07-3.38)
