# Supplementary material for: ERAP/HLA-C and KIR Genetic Profile in Couples with Recurrent Implantation Failure
Source: Int J Mol Sci. 2022 Oct 19;23(20):12518. doi: 10.3390/ijms232012518 (PMC9603896; doi:10.3390/ijms232012518)
Supplement: Supplementary file 1 [file ijms-23-12518-s001.zip › Supplementary Table S3.pdf]

Supplementary Table S3. Frequency of female *ERAP* and *KIR* with male *HLA-C* gene combinations in couples undergoing *in vitro* fertilization and in fertile couples.

Female ERAP/male HLA-C /female KIR	IVF	RIF	SIVF	Fertile
ERAP1 rs30187/ HLA-C/KIR	N = 115 (%)	N = 70 (%)	N = 30 (%)	N = 80 (%)
CC/C1+/AA	46 (40.00)	27 (38.57)	14 (46.67)	36 (45.00)
CT/C1+/AA	49 (42.61)	28 (40.00)	14 (46.67)	35 (43.75)
TT/C1+/AA	20 (17.39)	15 (21.43)	2 (6.67)	9 (11.25)
	N = 304 (%)	N = 166 (%)	N = 106 (%)	N = 192 (%)
CC/C1+/Bx	136 (44.74)	66 (39.76)	50 (47.17)	92 (47.92)
CT/C1+/Bx	139 (45.72)	85 (51.20)	43 (40.57)	87 (45.31)
TT/C1+/Bx	29 (9.54)	15 (9.04)	13 (12.26)	13 (6.77)
	N = 103 (%)	N = 54 (%)	N = 38 (%)	N = 66 (%)
CC/C2+/AA	38 (36.89)	19 (35.19)	16 (42.11)	33 (50.00)
CT/C2+/AA	50 (48.54)	24 (44.44)	20 (52.63)	26 (39.39)
TT/C2+/AA	15 (14.56)	11 (20.37)	2 (5.26)	7 (10.61)
	N = 217 (%)	N = 126 (%)	N = 72 (%)	N = 144 (%)
CC/C2+/Bx	103 (47.47)	55 (43.65)	37 (51.39)	73 (50.69)
CT/C2+/Bx	89 (41.01)	56 (44.44)	27 (37.50)	63 (43.75)
TT/C2+/Bx	25 (11.52)	15 (11.90)	8 (11.11)	8 (5.56)
	N = 35 (%)	N = 23 (%)	N = 6 (%)	N = 28 (%)
CC/C1C1/AA	16 (45.71)	10 (43.48)	3 (50.00)	11 (39.29)
CT/C1C1/AA	13 (37.14)	9 (39.13)	2 (33.33)	12 (42.86)
TT/C1C1/AA	6 (17.14)	4 (17.39)	1 (16.67)	5 (17.86)
	N = 80 (%)	N = 47 (%)	N = 24 (%)	N = 52 (%)
CC/C1C2/AA	30 (37.50)	17 (36.17)	11 (45.83)	25 (48.08)
CT/C1C2/AA	36 (45.00)	19 (40.43)	12 (50.00)	23 (44.23)
TT/C1C2/AA	14 (17.50)	11 (23.40)[a,b]	1 (4.17)	4 (7.69)
	N = 23 (%)	N = 7 (%)	N = 14 (%)	N = 14 (%)
CC/C2C2/AA	8 (34.78)	2 (28.57)	5 (35.71)	8 (57.14)
CT/C2C2/AA	14 (60.87)[c]	5 (71.43)	8 (57.14)	3 (21.43)
TT/C2C2/AA	1 (4.35)	0 (0.00)	1 (7.14)	3 (21.43)
	N = 136 (%)	N = 75 (%)	N = 45 (%)	N = 84 (%)
CC/C1C1/Bx	63 (46.32)	33 (44.00)	19 (42.22)	35 (41.67)
CT/C1C1/Bx	63 (46.32)	37 (49.33)	21 (46.67)	41 (48.81)
TT/C1C1/Bx	10 (7.35)	5 (6.67)	5 (11.11)	8 (9.52)
	N = 168 (%)	N = 91 (%)	N = 61 (%)	N = 108 (%)
CC/C1C2/Bx	73 (43.45)	33 (36.26)[d]	31 (50.82)	57 (52.78)
CT/C1C2/Bx	76 (45.24)	48 (52.75)[e]	22 (36.07)	46 (42.59)
TT/C1C2/Bx	19 (11.31)	10 (10.99)	8 (13.11)	5 (4.63)
	N = 49 (%)	N = 35 (%)	N = 11 (%)	N = 36 (%)
CC/C2C2/Bx	30 (61.22)	22 (62.86)	6 (54.55)	16 (44.44)
CT/C2C2/Bx	13 (26.53)	8 (22.86)[f]	5 (45.45)	17 (47.22)
TT/C2C2/Bx	6 (12.24)	5 (14.29)	0 (0.00)	3 (8.33)
ERAP1 rs27044/HLA-C/KIR	N = 115 (%)	N = 70 (%)	N = 30 (%)	N = 80 (%)
CC/C1+/AA	57 (49.57)	35 (50.00)	16 (53.33)	45 (56.25)

Female ERAP/male HLA-C /female KIR	IVF	RIF	SIVF	Fertile
CG/C1+/AA	43 (37.39)	24 (34.29)	13 (43.33)	27 (33.75)
GG/C1+/AA	15 (13.04)	11 (15.71)	1 (3.33)	8 (10.00)
	N = 304 (%)	N = 166 (%)	N = 106 (%)	N = 192 (%)
CC/C1+/Bx	162 (53.29)	**79 (47.59)**[g]	58 (54.72)	114 (59.38)
CG/C1+/Bx	126 (41.45)	**80 (48.19)**[h]	39 (36.79)	68 (35.42)
GG/C1+/Bx	16 (5.26)	7 (4.22)	9 (8.49)	10 (5.21)
	N = 103 (%)	N = 54 (%)	N = 38 (%)	N = 66 (%)
CC/C2+/AA	48 (46.60)	24 (44.44)	20 (52.63)	39 (59.09)
CG/C2+/AA	44 (42.72)	22 (40.74)	17 (44.74)	21 (31.82)
GG/C2+/AA	11 (10.68)	8 (14.81)	1 (2.63)	6 (9.09)
	N = 217 (%)	N = 126 (%)	N = 72 (%)	N = 144 (%)
CC/C2+/Bx	118 (54.38)	**63 (50.00)**[i]	39 (54.17)	91 (63.19)
CG/C2+/Bx	85 (39.17)	54 (42.86)	28 (38.89)	45 (31.25)
GG/C2+/Bx	14 (6.45)	9 (7.14)	5 (6.94)	8 (5.56)
	N = 35 (%)	N = 23 (%)	N = 6 (%)	N = 28 (%)
CC/C1C1/AA	20 (57.14)	13 (56.52)	4 (66.67)	15 (53.57)
CG/C1C1/AA	11 (31.43)	7 (30.43)	2 (33.33)	9 (32.14)
GG/C1C1/AA	4 (11.43)	3 (13.04)	0 (0.00)	4 (14.29)
	N = 80 (%)	N = 47 (%)	N = 24 (%)	N = 52 (%)
CC/C1C2/AA	37 (46.25)	22 (46.81)	12 (50.00)	30 (57.69)
CG/C1C2/AA	32 (40.00)	17 (36.17)	11 (45.83)	18 (34.62)
GG/C1C2/AA	11 (13.75)	8 (17.02)	1 (4.17)	4 (7.69)
	N = 23 (%)	N = 7 (%)	N = 14 (%)	N = 14 (%)
CC/C2C2/AA	11 (47.83)	2 (28.57)	8 (57.14)	9 (64.29)
CG/C2C2/AA	12 (52.17)	5 (71.43)	6 (42.86)	3 (21.43)
GG/C2C2/AA	0 (0.00)	0 (0.00)	0 (0.00)	2 (14.29)
	N = 136 (%)	N = 75 (%)	N = 45 (%)	N = 84 (%)
CC/C1C1/Bx	76 (55.88)	39 (52.00)	25 (55.56)	44 (52.38)
CG/C1C1/Bx	54 (39.71)	34 (45.33)	16 (35.56)	35 (41.67)
GG/C1C1/Bx	6 (4.41)	2 (2.67)	4 (8.89)	5 (5.95)
	N = 168 (%)	N = 91 (%)	N = 61 (%)	N = 108 (%)
CC/C1C2/Bx	**86 (51.19)**[j]	**40 (43.96)**[k]	33 (54.10)	70 (64.81)
CG/C1C2/Bx	**72 (42.86)**[l]	**46 (50.55)**[m]	23 (37.70)	33 (30.56)
GG/C1C2/Bx	10 (5.95)	5 (5.49)	5 (8.20)	5 (4.63)
	N = 49 (%)	N = 35 (%)	N = 11 (%)	N = 36 (%)
CC/C2C2/Bx	32 (65.31)	23 (65.71)	6 (54.55)	21 (58.33)
CG/C2C2/Bx	13 (26.53)	8 (22.86)	5 (45.45)	12 (33.33)
GG/C2C2/Bx	4 (8.16)	4 (11.43)	0 (0.00)	3 (8.33)
ERAP1 rs26653/HLA-C/KIR	N = 115 (%)	N = 70 (%)	N = 30 (%)	N = 79 (%)
GG/C1+/AA	63 (54.78)	35 (50.00)	18 (60.00)	48 (60.76)
CG/C1+/AA	41 (35.65)	30 (42.86)	7 (23.33)	27 (34.18)
CC/C1+/AA	11 (9.57)	5 (7.14)	5 (16.67)	4 (5.06)
	N = 304 (%)	N = 166 (%)	N = 106 (%)	N = 190 (%)
GG/C1+/Bx	168 (55.26)	88 (53.01)	61 (57.55)	100 (52.63)
CG/C1+/Bx	127 (41.78)	73 (43.98)	44 (41.51)	79 (41.58)
CC/C1+/Bx	9 (2.96)	5 (3.01)	1 (0.94)	11 (5.79)

Female ERAP/male HLA-C /female KIR	IVF	RIF	SIVF	Fertile
	N = 103 (%)	N = 54 (%)	N = 38 (%)	N = 65 (%)
GG/C2+/AA	55 (53.40)	27 (50.00)	22 (57.89)	42 (64.62)
CG/C2+/AA	41 (39.81)	24 (44.44)	13 (34.21)	20 (30.77)
CC/C2+/AA	7 (6.80)	3 (5.56)	3 (7.89)	3 (4.62)
	N = 217 (%)	N = 126 (%)	N = 72 (%)	N = 143 (%)
GG/C2+/Bx	123 (56.68)	69 (54.76)	43 (59.72)	79 (55.24)
CG/C2+/Bx	84 (38.71)	51 (40.48)	29 (40.28)	58 (40.56)
CC/C2+/Bx	10 (4.61)	6 (4.76)	0 (0.00)	6 (4.20)
	N = 35 (%)	N = 23 (%)	N = 6 (%)	N = 28 (%)
GG/C1C1/AA	16 (45.71)	10 (43.48)	1 (16.67)	16 (57.14)
CG/C1C1/AA	15 (42.86)	11 (47.83)	3 (50.00)	10 (35.71)
CC/C1C1/AA	4 (11.43)	2 (8.70)	2 (33.33)	2 (7.14)
	N = 80 (%)	N = 47 (%)	N = 24 (%)	N = 51 (%)
GG/C1C2/AA	47 (58.75)	25 (53.19)	17 (70.83)	32 (62.75)
CG/C1C2/AA	26 (32.50)	19 (40.43)	4 (16.67)	17 (33.33)
CC/C1C2/AA	7 (8.75)	3 (6.38)	3 (12.50)	2 (3.92)
	N = 23 (%)	N = 7 (%)	N = 14 (%)	N = 14 (%)
GG/C2C2/AA	**8 (34.78)**[n]	2 (28.57)	5 (35.71)	10 (71.43)
CG/C2C2/AA	**15 (65.22)**[o]	5 (71.43)	9 (64.29)	3 (21.43)
CC/C2C2/AA	0 (0.00)	0 (0.00)	0 (0.00)	1 (7.14)
	N = 136 (%)	N = 75 (%)	N = 45 (%)	N = 83 (%)
GG/C1C1/Bx	73 (53.68)	40 (53.33)	23 (51.11)	42 (50.60)
CG/C1C1/Bx	59 (43.38)	32 (42.67)	21 (46.67)	35 (42.17)
CC/C1C1/Bx	4 (2.94)	3 (4.00)	1 (2.22)	6 (7.23)
	N = 168 (%)	N = 91 (%)	N = 61 (%)	N = 107 (%)
GG/C1C2/Bx	95 (56.55)	48 (52.75)	38 (62.30)	58 (54.21)
CG/C1C2/Bx	68 (40.48)	41 (45.05)	23 (37.70)	44 (41.12)
CC/C1C2/Bx	5 (2.98)	2 (2.20)	0 (0.00)	5 (4.67)
	N = 49 (%)	N = 35 (%)	N = 11 (%)	N = 36 (%)
GG/C2C2/Bx	28 (57.14)	21 (60.00)	5 (45.45)	21 (58.33)
CG/C2C2/Bx	16 (32.65)	10 (28.57)	6 (54.55)	14 (38.89)
CC/C2C2/Bx	5 (10.20)	4 (11.43)	0 (0.00)	1 (2.78)
ERAP1 rs26618/HLA-C/KIR	N = 115 (%)	N = 70 (%)	N = 30 (%)	N = 80 (%)
TT/C1+/AA	67 (58.26)	40 (57.14)	18 (60.00)	38 (47.50)
CT/C1+/AA	42 (36.52)	25 (35.71)	11 (36.67)	32 (40.00)
CC/C1+/AA	6 (5.22)	5 (7.14)	1 (3.33)	10 (12.50)
	N = 304 (%)	N = 166 (%)	N = 106 (%)	N = 192 (%)
TT/C1+/Bx	154 (50.66)	92 (55.42)	49 (46.23)	91 (47.40)
CT/C1+/Bx	123 (40.46)	63 (37.95)	44 (41.51)	88 (45.83)
CC/C1+/Bx	27 (8.88)	11 (6.63)	13 (12.26)	13 (6.77)
	N = 103 (%)	N = 54 (%)	N = 38 (%)	N = 66 (%)
TT/C2+/AA	57 (55.34)	31 (57.41)	22 (57.89)	27 (40.91)
CT/C2+/AA	42 (40.78)	20 (37.04)	15 (39.47)	30 (45.45)
CC/C2+/AA	**4 (3.88)**[p]	3 (5.56)	1 (2.63)	9 (13.64)
	N = 217 (%)	N = 126 (%)	N = 72 (%)	N = 144 (%)
TT/C2+/Bx	114 (52.53)	**75 (59.52)**[q]	29 (40.28)	74 (51.39)

Female ERAP/male HLA-C /female KIR	IVF	RIF	SIVF	Fertile
CT/C2+/Bx	82 (37.79)	42 (33.33)	33 (45.83)	60 (41.67)
CC/C2+/Bx	21 (9.68)	9 (7.14)	10 (13.89)	10 (6.94)
	N = 35 (%)	N = 23 (%)	N = 6 (%)	N = 28 (%)
TT/C1C1/AA	22 (62.86)	13 (56.52)	4 (66.67)	17 (60.71)
CT/C1C1/AA	11 (31.43)	8 (34.78)	2 (33.33)	7 (25.00)
CC/C1C1/AA	2 (5.71)	2 (8.70)	0 (0.00)	4 (14.29)
	N = 80 (%)	N = 47 (%)	N = 24 (%)	N = 52 (%)
TT/C1C2/AA	45 (56.25)	27 (57.45)	14 (58.33)	21 (40.38)
CT/C1C2/AA	31 (38.75)	17 (36.17)	9 (37.50)	25 (48.08)
CC/C1C2/AA	4 (5.00)	3 (6.38)	1 (4.17)	6 (11.54)
	N = 23 (%)	N = 7 (%)	N = 14 (%)	N = 14 (%)
TT/C2C2/AA	12 (52.17)	4 (57.14)	8 (57.14)	6 (42.86)
CT/C2C2/AA	11 (47.83)	3 (42.86)	6 (42.86)	5 (35.71)
CC/C2C2/AA	0 (0.00)[r]	0 (0.00)	0 (0.00)	3 (21.43)
	N = 136 (%)	N = 75 (%)	N = 45 (%)	N = 84 (%)
TT/C1C1/Bx	71 (52.21)	40 (53.33)	26 (57.78)	37 (44.05)
CT/C1C1/Bx	53 (38.97)	29 (38.67)	15 (33.33)	43 (51.19)
CC/C1C1/Bx	12 (8.82)	6 (8.00)	4 (8.89)	4 (4.76)
	N = 168 (%)	N = 91 (%)	N = 61 (%)	N = 108 (%)
TT/C1C2/Bx	83 (49.40)	52 (57.14)[s]	23 (37.70)	54 (50.00)
CT/C1C2/Bx	70 (41.67)	34 (37.36)	29 (47.54)	45 (41.67)
CC/C1C2/Bx	15 (8.93)	5 (5.49)	9 (14.75)	9 (8.33)
	N = 49 (%)	N = 35 (%)	N = 11 (%)	N = 36 (%)
TT/C2C2/Bx	31 (63.27)	23 (65.71)	6 (54.55)	20 (55.56)
CT/C2C2/Bx	12 (24.49)	8 (22.86)	4 (36.36)	15 (41.67)
CC/C2C2/Bx	6 (12.24)	4 (11.43)	1 (9.09)	1 (2.78)
ERAP1 rs2287987/HLA-C/KIR	N = 115 (%)	N = 70 (%)	N = 30 (%)	N = 80 (%)
TT/C1+/AA	71 (61.74)	45 (64.29)	17 (56.67)	53 (66.25)
CT/C1+/AA	41 (35.65)	24 (34.29)	11 (36.67)	26 (32.50)
CC/C1+/AA	3 (2.61)	1 (1.43)	2 (6.67)	1 (1.25)
	N = 304 (%)	N = 166 (%)	N = 106 (%)	N = 192 (%)
TT/C1+/Bx	190 (62.50)	100 (60.24)	73 (68.87)	116 (60.42)
CT/C1+/Bx	98 (32.24)	57 (34.34)	28 (26.42)	70 (36.46)
CC/C1+/Bx	16 (5.26)	9 (5.42)	5 (4.72)	6 (3.12)
	N = 103 (%)	N = 54 (%)	N = 38 (%)	N = 66 (%)
TT/C2+/AA	68 (66.02)	37 (68.52)	23 (60.53)	44 (66.67)
CT/C2+/AA	32 (31.07)	16 (29.63)	13 (34.21)	21 (31.82)
CC/C2+/AA	3 (2.91)	1 (1.85)	2 (5.26)	1 (1.52)
	N = 217 (%)	N = 126 (%)	N = 72 (%)	N = 144 (%)
TT/C2+/Bx	133 (61.29)	77 (61.11)	46 (63.89)	78 (54.17)
CT/C2+/Bx	70 (32.26)	41 (32.54)	22 (30.56)	61 (42.36)
CC/C2+/Bx	14 (6.45)	8 (6.35)	4 (5.56)	5 (3.47)
	N = 35 (%)	N = 23 (%)	N = 6 (%)	N = 28 (%)
TT/C1C1/AA	21 (60.00)	15 (65.22)	4 (66.67)	20 (71.43)
CT/C1C1/AA	14 (40.00)	8 (34.78)	2 (33.33)	8 (28.57)
CC/C1C1/AA	0 (0.00)	0 (0.00)	0 (0.00)	0 (0.00)

Female ERAP/male HLA-C /female KIR	IVF	RIF	SIVF	Fertile
	N = 80 (%)	N = 47 (%)	N = 24 (%)	N = 52 (%)
TT/C1C2/AA	50 (62.50)	30 (63.83)	13 (54.17)	33 (63.46)
CT/C1C2/AA	27 (33.75)	16 (34.04)	9 (37.50)	18 (34.62)
CC/C1C2/AA	3 (3.75)	1 (2.13)	2 (8.33)	1 (1.92)
	N = 23 (%)	N = 7 (%)	N = 14 (%)	N = 14 (%)
TT/C2C2/AA	18 (78.26)	7 (100.00)	10 (71.43)	11 (78.57)
CT/C2C2/AA	5 (21.74)	0 (0.00)	4 (28.57)	3 (21.43)
CC/C2C2/AA	0 (0.00)	0 (0.00)	0 (0.00)	0 (0.00)
	N = 136 (%)	N = 75 (%)	N = 45 (%)	N = 84 (%)
TT/C1C1/Bx	82 (60.29)	43 (57.33)	30 (66.67)	56 (66.67)
CT/C1C1/Bx	47 (34.56)	27 (36.00)	13 (28.89)	26 (30.95)
CC/C1C1/Bx	7 (5.15)	5 (6.67)	2 (4.44)	2 (2.38)
	N = 168 (%)	N = 91 (%)	N = 61 (%)	N = 108 (%)
TT/C1C2/Bx	108 (64.29)	57 (62.64)	43 (70.49)	60 (55.56)
CT/C1C2/Bx	51 (30.36)	30 (32.97)	**15 (24.59)**[t]	44 (40.74)
CC/C1C2/Bx	9 (5.36)	4 (4.40)	3 (4.92)	4 (3.70)
	N = 49 (%)	N = 35 (%)	N = 11 (%)	N = 36 (%)
TT/C2C2/Bx	25 (51.02)	20 (57.14)	3 (27.27)	18 (50.00)
CT/C2C2/Bx	19 (38.78)	11 (31.43)	7 (63.64)	17 (47.22)
CC/C2C2/Bx	5 (10.20)	4 (11.43)	1 (9.09)	1 (2.78)
ERAP2 rs2248374/HLA-C/KIR	N = 114 (%)	N = 69 (%)	N = 30 (%)	N = 79 (%)
AA/C1+/AA	29 (25.44)	19 (27.54)	7 (23.33)	20 (25.32)
AG/C1+/AA	49 (42.98)	31 (44.93)	13 (43.33)	39 (49.37)
GG/C1+/AA	36 (31.58)	19 (27.54)	10 (33.33)	20 (25.32)
	N = 304 (%)	N = 166 (%)	N = 106 (%)	N = 190 (%)
AA/C1+/Bx	78 (25.66)	39 (23.49)	33 (31.13)	52 (27.37)
AG/C1+/Bx	149 (49.01)	80 (48.19)	49 (46.23)	94 (49.47)
GG/C1+/Bx	77 (25.33)	47 (28.31)	24 (22.64)	44 (23.16)
	N = 102 (%)	N = 53 (%)	N = 38 (%)	N = 65 (%)
AA/C2+/AA	22 (21.57)	11 (20.75)	9 (23.68)	16 (24.62)
AG/C2+/AA	49 (48.04)	25 (47.17)	20 (52.63)	35 (53.85)
GG/C2+/AA	31 (30.39)	17 (32.08)	9 (23.68)	14 (21.54)
	N = 217 (%)	N = 126 (%)	N = 72 (%)	N = 143 (%)
AA/C2+/Bx	60 (27.65)	**30 (23.81)**[u]	28 (38.89)	38 (26.57)
AG/C2+/Bx	105 (48.39)	60 (47.62)	30 (41.67)	65 (45.45)
GG/C2+/Bx	52 (23.96)	36 (28.57)	14 (19.44)	40 (27.97)
	N = 35 (%)	N = 23 (%)	N = 6 (%)	N = 28 (%)
AA/C1C1/AA	11 (31.43)	8 (34.78)	1 (16.67)	8 (28.57)
AG/C1C1/AA	15 (42.86)	11 (47.83)	3 (50.00)	12 (42.86)
GG/C1C1/AA	9 (25.71)	4 (17.39)	2 (33.33)	8 (28.57)
	N = 79 (%)	N = 46 (%)	N = 24 (%)	N = 51 (%)
AA/C1C2/AA	18 (22.78)	11 (23.91)	6 (25.00)	12 (23.53)
AG/C1C2/AA	34 (43.04)	20 (43.48)	10 (41.67)	27 (52.94)
GG/C1C2/AA	27 (34.18)	15 (32.61)	8 (33.33)	12 (23.53)
	N = 23 (%)	N = 7 (%)	N = 14 (%)	N = 14 (%)
AA/C2C2/AA	4 (17.39)	0 (0.00)	3 (21.43)	4 (28.57)

Female ERAP/male HLA-C /female KIR	IVF	RIF	SIVF	Fertile
AG/C2C2/AA	15 (65.22)	5 (71.43)	10 (71.43)	8 (57.14)
GG/C2C2/AA	4 (17.39)	2 (28.57)	1 (7.14)	2 (14.29)
	N = 136 (%)	N = 75 (%)	N = 45 (%)	N = 83 (%)
AA/C1C1/Bx	33 (24.26)	18 (24.00)	10 (22.22)	24 (28.92)
AG/C1C1/Bx	66 (48.53)	36 (48.00)	23 (51.11)	42 (50.60)
GG/C1C1/Bx	37 (27.21)	21 (28.00)	12 (26.67)	17 (20.48)
	N = 168 (%)	N = 91 (%)	N = 61 (%)	N = 107 (%)
AA/C1C2/Bx	45 (26.79)	21 (23.08)	23 (37.70)	28 (26.17)
AG/C1C2/Bx	83 (49.40)	44 (48.35)	26 (42.62)	52 (48.60)
GG/C1C2/Bx	40 (23.81)	26 (28.57)	12 (19.67)	27 (25.23)
	N = 49 (%)	N = 35 (%)	N = 11 (%)	N = 36 (%)
AA/C2C2/Bx	15 (30.61)	9 (25.71)	5 (45.45)	10 (27.78)
AG/C2C2/Bx	22 (44.90)	16 (45.71)	4 (36.36)	13 (36.11)
GG/C2C2/Bx	12 (24.49)	10 (28.57)	2 (18.18)	13 (36.11)
ERAP1 rs6861666/HLA-C/KIR	N = 114 (%)	N = 69 (%)	N = 30 (%)	N = 79 (%)
AA/C1+/AA	97 (85.09)	60 (86.96)	24 (80.00)	69 (87.34)
AG/C1+/AA	16 (14.04)	8 (11.59)	6 (20.00)	10 (12.66)
GG/C1+/AA	1 (0.88)	1 (1.45)	0 (0.00)	0 (0.00)
	N = 304 (%)	N = 166 (%)	N = 106 (%)	N = 188 (%)
AA/C1+/Bx	261 (85.86)	138 (83.13)	92 (86.79)	160 (85.11)
AG/C1+/Bx	42 (13.82)	28 (16.87)	13 (12.26)	28 (14.89)
GG/C1+/Bx	1 (0.33)	0 (0.00)	1 (0.94)	0 (0.00)
	N = 102 (%)	N = 53 (%)	N = 38 (%)	N = 65 (%)
AA/C2+/AA	87 (85.29)	47 (88.68)	32 (84.21)	56 (86.15)
AG/C2+/AA	15 (14.71)	6 (11.32)	6 (15.79)	9 (13.85)
GG/C2+/AA	0 (0.00)	0 (0.00)	0 (0.00)	0 (0.00)
	N = 217 (%)	N = 126 (%)	N = 72 (%)	N = 142 (%)
AA/C2+/Bx	188 (86.64)	108 (85.71)	62 (86.11)	118 (83.10)
AG/C2+/Bx	29 (13.36)	18 (14.29)	10 (13.89)	23 (16.20)
GG/C2+/Bx	0 (0.00)	0 (0.00)	0 (0.00)	1 (0.70)
	N = 35 (%)	N = 23 (%)	N = 6 (%)	N = 28 (%)
AA/C1C1/AA	28 (80.00)	18 (78.26)	4 (66.67)	26 (92.86)
AG/C1C1/AA	6 (17.14)	4 (17.39)	2 (33.33)	2 (7.14)
GG/C1C1/AA	1 (2.86)	1 (4.35)	0 (0.00)	0 (0.00)
	N = 79 (%)	N = 46 (%)	N = 24 (%)	N = 51 (%)
AA/C1C2/AA	69 (87.34)	42 (91.30)	20 (83.33)	43 (84.31)
AG/C1C2/AA	10 (12.66)	4 (8.70)	4 (16.67)	8 (15.69)
GG/C1C2/AA	0 (0.00)	0 (0.00)	0 (0.00)	0 (0.00)
	N = 23 (%)	N = 7 (%)	N = 14 (%)	N = 14 (%)
AA/C2C2/AA	18 (78.26)	5 (71.43)	12 (85.71)	13 (92.86)
AG/C2C2/AA	5 (21.74)	2 (28.57)	2 (14.29)	1 (7.14)
GG/C2C2/AA	0 (0.00)	0 (0.00)	0 (0.00)	0 (0.00)
	N = 136 (%)	N = 75 (%)	N = 45 (%)	N = 82 (%)
AA/C1C1/Bx	117 (86.03)	61 (81.33)	40 (88.89)	68 (82.93)
AG/C1C1/Bx	18 (13.24)	14 (18.67)	4 (8.89)	14 (17.07)
GG/C1C1/Bx	1 (0.74)	0 (0.00)	1 (2.22)	0 (0.00)

Female ERAP/male HLA-C /female KIR	IVF	RIF	SIVF	Fertile
	N = 168 (%)	N = 91 (%)	N = 61 (%)	N = 106 (%)
AA/C1C2/Bx	144 (85.71)	77 (84.62)	52 (85.25)	92 (86.79)
AG/C1C2/Bx	24 (14.29)	14 (15.38)	9 (14.75)	14 (13.21)
GG/C1C2/Bx	0 (0.00)	0 (0.00)	0 (0.00)	0 (0.00)
	N = 49 (%)	N = 35 (%)	N = 11 (%)	N = 36 (%)
AA/C2C2/Bx	**44 (89.80)**[w]	31 (88.57)	10 (90.91)	26 (72.22)
AG/C2C2/Bx	5 (10.20)	4 (11.43)	1 (9.09)	9 (25.00)
GG/C2C2/Bx	0 (0.00)	0 (0.00)	0 (0.00)	1 (2.78)

IVF-ET – in vitro fertilization embryo transfer; RIF – recurrent implantation failure; SIVF – successful pregnancy after IVF-ET; p – probability; $p_{corr.}$ – probability after Bonferroni correction for multiple comparisons (x6 for possible *ERAP* with *HLA-C* C1+ or C2+ and *KIR* AA or Bx combinations; x9 for possible genotypes *ERAP*, *HLA-C* with *KIR* AA or Bx combinations); OR – odds ratio; 95% CI – confidence interval from two-sided Fisher's exact test; ns – not significant. Values in bold indicate significant differences.

IVF *vs.* Fertile: [c]p/$p_{corr.}$ = 0.040/ns, OR = 5.425, 95% CI (1.04-38.86); [j]p/$p_{corr.}$ = 0.034/ns, OR = 0.570, 95% CI (0.33-0.96); [l]p/$p_{corr.}$ = 0.043/ns, OR = 1.701, 95% CI (0.99-2.95); [n]p/$p_{corr.}$ = 0.045/ns, OR = 0.223, 95% CI (0.04-1.09); [o]p/$p_{corr.}$ = 0.017/ns, OR = 6.490, 95% CI (1.23-47.02); [p]p/$p_{corr.}$ = 0.035/ns, OR = 0.258, 95% CI (0.06-0.98); [r]p/$p_{corr.}$ = 0.047/ns, OR = 0.000, 95% CI (0.00-1.38); [w]p/$p_{corr.}$ = 0.046/ns, OR = 3.334, 95% CI (0.92-13.86);

RIF *vs.* Fertile: [a]p/$p_{corr.}$ = 0.047/ns, OR = 3.620, 95% CI (0.97-16.88); [d]p/$p_{corr.}$ = 0.023/ns, OR = 0.511, 95% CI (0.28-0.94); [f]p/$p_{corr.}$ = 0.047/ns, OR = 0.337, 95% CI (0.10-1.03); [g]p/$p_{corr.}$ = 0.033/ns, OR = 0.622, 95% CI (0.40-0.97); [h]p/$p_{corr.}$ = 0.018/ns, OR = 1.694, 95% CI (1.09-2.65); [i]p/$p_{corr.}$ = 0.036/ns, OR = 0.584, 95% CI (0.35-0.98). [k]p/$p_{corr.}$ = 0.004/0.037, OR = 0.428, 95% CI (0.23-0.78); [m]p/$p_{corr.}$ = 0.006/0.050, OR = 2.313, 95% CI (1.25-4.33);

SIVF *vs.* Fertile: [t]p/$p_{corr.}$ = 0.044/ns, OR = 0.476, 95% CI (0.22-1.00);

RIF *vs.* SIVF: [b]p/$p_{corr.}$ = 0.049/ns, OR = 6.886, 95% CI (0.89-314.94); [e]p/$p_{corr.}$ = 0.048/ns, OR = 1.970, 95% CI (0.97-4.08); [q]p/$p_{corr.}$ = 0.012/ns, OR = 2.172, 95% CI (1.16-4.11); [s]p/$p_{corr.}$ = 0.021/ns, OR = 2.191, 95% CI (1.08-4.52); [u]p/$p_{corr.}$ = 0.034/ns, OR = 0.493, 95% CI (0.25-0.97)
