# Supplementary material for: ERAP/HLA-C and KIR Genetic Profile in Couples with Recurrent Implantation Failure
Source: Int J Mol Sci. 2022 Oct 19;23(20):12518. doi: 10.3390/ijms232012518 (PMC9603896; doi:10.3390/ijms232012518)
Supplement: Supplementary file 1 [file ijms-23-12518-s001.zip › Supplementary Table S4.pdf]

Supplementary Table S4. Distribution of female *KIR/HLA-C* with her partner's *ERAP* genotype combinations in couples undergoing *in vitro* fertilization and in fertile couples.

Male ERAP/female HLA-C/ female KIR	IVF	RIF	SIVF	Fertile
ERAP1 rs30187/HLA-C/KIR	N = 108 (%)	N = 58 (%)	N = 36 (%)	N = 82 (%)
CC/C1+/AA	61 (56.48)	37 (63.79)	17 (47.22)	40 (48.78)
CT/C1+/AA	38 (35.19)	19 (32.76)	12 (33.33)	30 (36.59)
TT/C1+/AA	9 (8.33)	2 (3.45)[a.b]	7 (19.44)	12 (14.63)
	N = 296 (%)	N = 170 (%)	N = 98 (%)	N = 190 (%)
CC/C1+/Bx	151 (51.01)	84 (49.41)	51 (52.04)	82 (43.16)
CT/C1+/Bx	115 (38.85)	68 (40.00)	37 (37.76)	90 (47.37)
TT/C1+/Bx	30 (10.14)	18 (10.59)	10 (10.20)	18 (9.47)
	N = 88 (%)	N = 48 (%)	N = 30 (%)	N = 52 (%)
CC/C2+/AA	42 (47.73)	24 (50.00)	15 (50.00)	25 (48.08)
CT/C2+/AA	36 (40.91)	20 (41.67)	9 (30.00)	22 (42.31)
TT/C2+/AA	10 (11.36)	4 (8.33)	6 (20.00)	5 (9.62)
	N = 242 (%)	N = 136 (%)	N = 79 (%)	N = 149 (%)
CC/C2+/Bx	117 (48.35)	66 (48.53)	38 (48.10)	63 (42.28)
CT/C2+/Bx	99 (40.91)	51 (37.50)	35 (44.30)	70 (46.98)
TT/C2+/Bx	26 (10.74)	19 (13.97)	6 (7.59)	16 (10.74)
	N = 49 (%)	N = 29 (%)	N = 14 (%)	N = 40 (%)
CC/C1C1/AA	30 (61.22)	20 (68.97)	6 (42.86)	19 (47.50)
CT/C1C1/AA	14 (28.57)	8 (27.59)	4 (28.57)	13 (32.50)
TT/C1C1/AA	5 (10.20)	1 (3.45)	4 (28.57)	8 (20.00)
	N = 59 (%)	N = 29 (%)	N = 22 (%)	N = 42 (%)
CC/C1C2/AA	31 (52.54)	17 (58.62)	11 (50.00)	21 (50.00)
CT/C1C2/AA	24 (40.68)	11 (37.93)	8 (36.36)	17 (40.48)
TT/C1C2/AA	4 (6.78)	1 (3.45)	3 (13.64)	4 (9.52)
	N = 29 (%)	N = 19 (%)	N = 8 (%)	N = 10 (%)
CC/C2C2/AA	11 (37.93)	7 (36.84)	4 (50.00)	4 (40.00)
CT/C2C2/AA	12 (41.38)	9 (47.37)[c]	1 (12.50)	5 (50.00)
TT/C2C2/AA	6 (20.69)	3 (15.79)	3 (37.50)	1 (10.00)
	N = 111 (%)	N = 65 (%)	N = 38 (%)	N = 76 (%)
CC/C1C1/Bx	59 (53.15)	32 (49.23)	22 (57.89)	36 (47.37)
CT/C1C1/Bx	44 (39.64)	30 (46.15)	12 (31.58)	34 (44.74)
TT/C1C1/Bx	8 (7.21)	3 (4.62)	4 (10.53)	6 (7.89)
	N = 185 (%)	N = 105 (%)	N = 60 (%)	N = 114 (%)
CC/C1C2/Bx	92 (49.73)	52 (49.52)	29 (48.33)	46 (40.35)
CT/C1C2/Bx	71 (38.38)	38 (36.19)	25 (41.67)	56 (49.12)
TT/C1C2/Bx	22 (11.89)	15 (14.29)	6 (10.00)	12 (10.53)
	N = 57 (%)	N = 31 (%)	N = 19 (%)	N = 35 (%)
CC/C2C2/Bx	25 (43.86)	14 (45.16)	9 (47.37)	17 (48.57)
CT/C2C2/Bx	28 (49.12)	13 (41.94)	10 (52.63)	14 (40.00)
TT/C2C2/Bx	4 (7.02)	4 (12.90)	0 (0.00)	4 (11.43)
ERAP1 rs27044/HLA-C/KIR	N = 108 (%)	N = 58 (%)	N = 36 (%)	N = 82 (%)
CC/C1+/AA	68 (62.96)	42 (72.41)	17 (47.22)	49 (59.76)

Male ERAP/female HLA-C/ female KIR	IVF	RIF	SIVF	Fertile
CG/C1+/AA	33 (30.56)	15 (25.86)	13 (36.11)	27 (32.93)
GG/C1+/AA	7 (6.48)	**1 (1.72)**[d]	6 (16.67)	6 (7.32)
	N = 296 (%)	N = 170 (%)	N = 98 (%)	N = 190 (%)
CC/C1+/Bx	177 (59.80)	103 (60.59)	55 (56.12)	101 (53.16)
CG/C1+/Bx	98 (33.11)	57 (33.53)	34 (34.69)	74 (38.95)
GG/C1+/Bx	21 (7.09)	10 (5.88)	9 (9.18)	15 (7.89)
	N = 88 (%)	N = 48 (%)	N = 30 (%)	N = 52 (%)
CC/C2+/AA	49 (55.68)	29 (60.42)	15 (50.00)	29 (55.77)
CG/C2+/AA	30 (34.09)	16 (33.33)	9 (30.00)	19 (36.54)
GG/C2+/AA	9 (10.23)	3 (6.25)	6 (20.00)	4 (7.69)
	N = 242 (%)	N = 136 (%)	N = 79 (%)	N = 149 (%)
CC/C2+/Bx	136 (56.20)	78 (57.35)	42 (53.16)	77 (51.68)
CG/C2+/Bx	88 (36.36)	48 (35.29)	30 (37.97)	59 (39.60)
GG/C2+/Bx	18 (7.44)	10 (7.35)	7 (8.86)	13 (8.72)
	N = 49 (%)	N = 29 (%)	N = 14 (%)	N = 40 (%)
CC/C1C1/AA	32 (65.31)	22 (75.86)	6 (42.86)	24 (60.00)
CG/C1C1/AA	14 (28.57)	7 (24.14)	5 (35.71)	12 (30.00)
GG/C1C1/AA	3 (6.12)	0 (0.00)	3 (21.43)	4 (10.00)
	N = 59 (%)	N = 29 (%)	N = 22 (%)	N = 42 (%)
CC/C1C2/AA	36 (61.02)	20 (68.97)	11 (50.00)	25 (59.52)
CG/C1C2/AA	19 (32.20)	8 (27.59)	8 (36.36)	15 (35.71)
GG/C1C2/AA	4 (6.78)	1 (3.45)	3 (13.64)	2 (4.76)
	N = 29 (%)	N = 19 (%)	N = 8 (%)	N = 10 (%)
CC/C2C2/AA	13 (44.83)	9 (47.37)	4 (50.00)	4 (40.00)
CG/C2C2/AA	11 (37.93)	**8 (42.11)**[e]	1 (12.50)	4 (40.00)
GG/C2C2/AA	5 (17.24)	2 (10.53)	3 (37.50)	2 (20.00)
	N = 111 (%)	N = 65 (%)	N = 38 (%)	N = 76 (%)
CC/C1C1/Bx	70 (63.06)	41 (63.08)	23 (60.53)	46 (60.53)
CG/C1C1/Bx	37 (33.33)	23 (35.38)	13 (34.21)	24 (31.58)
GG/C1C1/Bx	4 (3.60)	1 (1.54)	2 (5.26)	6 (7.89)
	N = 185 (%)	N = 105 (%)	N = 60 (%)	N = 114 (%)
CC/C1C2/Bx	107 (57.84)	62 (59.05)	32 (53.33)	55 (48.25)
CG/C1C2/Bx	61 (32.97)	34 (32.38)	21 (35.00)	50 (43.86)
GG/C1C2/Bx	17 (9.19)	9 (8.57)	7 (11.67)	9 (7.89)
	N = 57 (%)	N = 31 (%)	N = 19 (%)	N = 35 (%)
CC/C2C2/Bx	29 (50.88)	16 (51.61)	10 (52.63)	22 (62.86)
CG/C2C2/Bx	**27 (47.37)**[f]	14 (45.16)	9 (47.37)	9 (25.71)
GG/C2C2/Bx	1 (1.75)	1 (3.23)	0 (0.00)	4 (11.43)
ERAP1 rs26653/HLA-C/KIR	N = 108 (%)	N = 58 (%)	N = 36 (%)	N = 82 (%)
GG/C1+/AA	66 (61.11)	34 (58.62)	22 (61.11)	43 (52.44)
CG/C1+/AA	35 (32.41)	23 (39.66)	9 (25.00)	28 (34.15)
CC/C1+/AA	7 (6.48)	**1 (1.72)**[g,h]	5 (13.89)	11 (13.41)
	N = 296 (%)	N = 170 (%)	N = 98 (%)	N = 190 (%)
GG/C1+/Bx	159 (53.72)	93 (54.71)	52 (53.06)	95 (50.00)
CG/C1+/Bx	113 (38.18)	61 (35.88)	39 (39.80)	86 (45.26)
CC/C1+/Bx	24 (8.11)	16 (9.41)	7 (7.14)	9 (4.74)

Male ERAP/female HLA-C/ female KIR	IVF	RIF	SIVF	Fertile
	N = 88 (%)	N = 48 (%)	N = 30 (%)	N = 52 (%)
GG/C2+/AA	54 (61.36)	28 (58.33)	20 (66.67)	30 (57.69)
CG/C2+/AA	26 (29.55)	17 (35.42)	6 (20.00)	14 (26.92)
CC/C2+/AA	8 (9.09)	3 (6.25)	4 (13.33)	8 (15.38)
	N = 242 (%)	N = 136 (%)	N = 79 (%)	N = 149 (%)
GG/C2+/Bx	136 (56.20)	76 (55.88)	45 (56.96)	75 (50.34)
CG/C2+/Bx	84 (34.71)	44 (32.35)	29 (36.71)	64 (42.95)
CC/C2+/Bx	22 (9.09)	16 (11.76)	5 (6.33)	10 (6.71)
	N = 49 (%)	N = 29 (%)	N = 14 (%)	N = 40 (%)
GG/C1C1/AA	28 (57.14)	16 (55.17)	7 (50.00)	18 (45.00)
CG/C1C1/AA	17 (34.69)	12 (41.38)	4 (28.57)	17 (42.50)
CC/C1C1/AA	4 (8.16)	1 (3.45)	3 (21.43)	5 (12.50)
	N = 59 (%)	N = 29 (%)	N = 22 (%)	N = 42 (%)
GG/C1C2/AA	38 (64.41)	18 (62.07)	15 (68.18)	25 (59.52)
CG/C1C2/AA	18 (30.51)	11 (37.93)	5 (22.73)	11 (26.19)
CC/C1C2/AA	3 (5.08)	0 (0.00)	2 (9.09)	6 (14.29)
	N = 29 (%)	N = 19 (%)	N = 8 (%)	N = 10 (%)
GG/C2C2/AA	16 (55.17)	10 (52.63)	5 (62.50)	5 (50.00)
CG/C2C2/AA	8 (27.59)	6 (31.58)[i]	1 (12.50)	3 (30.00)
CC/C2C2/AA	5 (17.24)	3 (15.79)	2 (25.00)	2 (20.00)
	N = 111 (%)	N = 65 (%)	N = 38 (%)	N = 76 (%)
GG/C1C1/Bx	58 (52.25)	35 (53.85)	20 (52.63)	39 (51.32)
CG/C1C1/Bx	45 (40.54)	25 (38.46)	15 (39.47)	34 (44.74)
CC/C1C1/Bx	8 (7.21)	5 (7.69)	3 (7.89)	3 (3.95)
	N = 185 (%)	N = 105 (%)	N = 60 (%)	N = 114 (%)
GG/C1C2/Bx	101 (54.59)	58 (55.24)	32 (53.33)	56 (49.12)
CG/C1C2/Bx	68 (36.76)	36 (34.29)	24 (40.00)	52 (45.61)
CC/C1C2/Bx	16 (8.65)	11 (10.48)	4 (6.67)	6 (5.26)
	N = 57 (%)	N = 31 (%)	N = 19 (%)	N = 35 (%)
GG/C2C2/Bx	35 (61.40)	18 (58.06)	13 (68.42)	19 (54.29)
CG/C2C2/Bx	16 (28.07)	8 (25.81)	5 (26.32)	12 (34.29)
CC/C2C2/Bx	6 (10.53)	5 (16.13)	1 (5.26)	4 (11.43)
ERAP1 rs26618/HLA-C/KIR	N = 108 (%)	N = 58 (%)	N = 36 (%)	N = 82 (%)
TT/C1+/AA	56 (51.85)	32 (55.17)	19 (52.78)	51 (62.20)
CT/C1+/AA	40 (37.04)	21 (36.21)	12 (33.33)	22 (26.83)
CC/C1+/AA	12 (11.11)	5 (8.62)	5 (13.89)	9 (10.98)
	N = 296 (%)	N = 170 (%)	N = 98 (%)	N = 190 (%)
TT/C1+/Bx	146 (49.32)	87 (51.18)	41 (41.84)	87 (45.79)
CT/C1+/Bx	122 (41.22)	70 (41.18)	45 (45.92)	89 (46.84)
CC/C1+/Bx	28 (9.46)	13 (7.65)	12 (12.24)	14 (7.37)
	N = 88 (%)	N = 48 (%)	N = 30 (%)	N = 51 (%)
TT/C2+/AA	43 (48.86)	23 (47.92)	16 (53.33)	28 (54.90)
CT/C2+/AA	37 (42.05)	20 (41.67)	11 (36.67)	13 (25.49)
CC/C2+/AA	8 (9.09)	5 (10.42)	3 (10.00)	10 (19.61)
	N = 242 (%)	N = 136 (%)	N = 79 (%)	N = 149 (%)
TT/C2+/Bx	123 (50.83)	71 (52.21)	35 (44.30)	76 (51.01)

Male ERAP/female HLA-C/ female KIR	IVF	RIF	SIVF	Fertile
CT/C2+/Bx	90 (37.19)	49 (36.03)	33 (41.77)	64 (42.95)
CC/C2+/Bx	29 (11.98)	16 (11.76)	**11 (13.92)**[j]	9 (6.04)
	N = 49 (%)	N = 29 (%)	N = 14 (%)	N = 40 (%)
TT/C1C1/AA	30 (61.22)	18 (62.07)	10 (71.43)	30 (75.00)
CT/C1C1/AA	13 (26.53)	9 (31.03)	2 (14.29)	10 (25.00)
CC/C1C1/AA	**6 (12.24)**[k]	2 (6.90)	2 (14.29)	0 (0.00)
	N = 59 (%)	N = 29 (%)	N = 22 (%)	N = 42 (%)
TT/C1C2/AA	26 (44.07)	14 (48.28)	9 (40.91)	21 (50.00)
CT/C1C2/AA	27 (45.76)	12 (41.38)	10 (45.45)	12 (28.57)
CC/C1C2/AA	6 (10.17)	3 (10.34)	3 (13.64)	9 (21.43)
	N = 29 (%)	N = 19 (%)	N = 8 (%)	N = 9 (%)
TT/C2C2/AA	17 (58.62)	9 (47.37)	7 (87.50)	7 (77.78)
CT/C2C2/AA	10 (34.48)	**8 (42.11)**[l]	1 (12.50)	1 (11.11)
CC/C2C2/AA	2 (6.90)	2 (10.53)	0 (0.00)	1 (11.11)
	N = 111 (%)	N = 65 (%)	N = 38 (%)	N = 76 (%)
TT/C1C1/Bx	55 (49.55)	32 (49.23)	17 (44.74)	33 (43.42)
CT/C1C1/Bx	50 (45.05)	31 (47.69)	18 (47.37)	36 (47.37)
CC/C1C1/Bx	6 (5.41)	2 (3.08)	3 (7.89)	7 (9.21)
	N = 185 (%)	N = 105 (%)	N = 60 (%)	N = 114 (%)
TT/C1C2/Bx	91 (49.19)	**55 (52.38)**[m]	24 (40.00)	54 (47.37)
CT/C1C2/Bx	72 (38.92)	39 (37.14)	27 (45.00)	53 (46.49)
CC/C1C2/Bx	22 (11.89)	11 (10.48)	9 (15.00)	7 (6.14)
	N = 57 (%)	N = 31 (%)	N = 19 (%)	N = 35 (%)
TT/C2C2/Bx	32 (56.14)	16 (51.61)	11 (57.89)	22 (62.86)
CT/C2C2/Bx	18 (31.58)	10 (32.26)	6 (31.58)	11 (31.43)
CC/C2C2/Bx	7 (12.28)	5 (16.13)	2 (10.53)	2 (5.71)
ERAP1 rs2287987/HLA-C/KIR	N = 108 (%)	N = 58 (%)	N = 36 (%)	N = 82 (%)
TT/C1+/AA	63 (58.33)	31 (53.45)	24 (66.67)	51 (62.20)
CT/C1+/AA	32 (29.63)	18 (31.03)	9 (25.00)	27 (32.93)
CC/C1+/AA	13 (12.04)	**9 (15.52)**[n]	3 (8.33)	4 (4.88)
	N = 295 (%)	N = 170 (%)	N = 97 (%)	N = 190 (%)
TT/C1+/Bx	191 (64.75)	110 (64.71)	65 (67.01)	118 (62.11)
CT/C1+/Bx	91 (30.85)	51 (30.00)	29 (29.90)	67 (35.26)
CC/C1+/Bx	13 (4.41)	9 (5.29)	3 (3.09)	5 (2.63)
	N = 88 (%)	N = 48 (%)	N = 30 (%)	N = 51 (%)
TT/C2+/AA	53 (60.23)	30 (62.50)	18 (60.00)	35 (68.63)
CT/C2+/AA	26 (29.55)	13 (27.08)	9 (30.00)	15 (29.41)
CC/C2+/AA	9 (10.23)	5 (10.42)	3 (10.00)	1 (1.96)
	N = 241 (%)	N = 136 (%)	N = 78 (%)	N = 149 (%)
TT/C2+/Bx	152 (63.07)	90 (66.18)	49 (62.82)	96 (64.43)
CT/C2+/Bx	78 (32.37)	39 (28.68)	27 (34.62)	48 (32.21)
CC/C2+/Bx	11 (4.56)	7 (5.15)	2 (2.56)	5 (3.36)
	N = 49 (%)	N = 29 (%)	N = 14 (%)	N = 40 (%)
TT/C1C1/AA	29 (59.18)	15 (51.72)	10 (71.43)	22 (55.00)
CT/C1C1/AA	13 (26.53)	8 (27.59)	3 (21.43)	15 (37.50)
CC/C1C1/AA	7 (14.29)	6 (20.69)	1 (7.14)	3 (7.50)

Male ERAP/female HLA-C/ female KIR	IVF	RIF	SIVF	Fertile
	N = 59 (%)	N = 29 (%)	N = 22 (%)	N = 42 (%)
TT/C1C2/AA	34 (57.63)	16 (55.17)	14 (63.64)	29 (69.05)
CT/C1C2/AA	19 (32.20)	10 (34.48)	6 (27.27)	12 (28.57)
CC/C1C2/AA	6 (10.17)	3 (10.34)	2 (9.09)	1 (2.38)
	N = 29 (%)	N = 19 (%)	N = 8 (%)	N = 9 (%)
TT/C2C2/AA	19 (65.52)	**14 (73.68)**[o]	4 (50.00)	6 (66.67)
CT/C2C2/AA	7 (24.14)	3 (15.79)	3 (37.50)	3 (33.33)
CC/C2C2/AA	3 (10.34)	2 (10.53)	1 (12.50)	0 (0.00)
	N = 111 (%)	N = 65 (%)	N = 38 (%)	N = 76 (%)
TT/C1C1/Bx	69 (62.16)	41 (63.08)	23 (60.53)	45 (59.21)
CT/C1C1/Bx	38 (34.23)	21 (32.31)	14 (36.84)	29 (38.16)
CC/C1C1/Bx	4 (3.60)	3 (4.62)	1 (2.63)	2 (2.63)
	N = 184 (%)	N = 105 (%)	N = 59 (%)	N = 114 (%)
TT/C1C2/Bx	122 (66.30)	69 (65.71)	42 (71.19)	73 (64.04)
CT/C1C2/Bx	53 (28.80)	30 (28.57)	15 (25.42)	38 (33.33)
CC/C1C2/Bx	9 (4.89)	6 (5.71)	2 (3.39)	3 (2.63)
	N = 57 (%)	N = 31 (%)	N = 19 (%)	N = 35 (%)
TT/C2C2/Bx	30 (52.63)	21 (67.74)	**7 (36.84)**[q]	23 (65.71)
CT/C2C2/Bx	25 (43.86)	**9 (29.03)**[p]	**12 (63.16)**[r]	10 (28.57)
CC/C2C2/Bx	2 (3.51)	1 (3.23)	0 (0.00)	2 (5.71)
ERAP2 rs2248374/HLA-C/KIR	N = 107 (%)	N = 58 (%)	N = 35 (%)	N = 82 (%)
AA/C1+/AA	33 (30.84)	17 (29.31)	12 (34.29)	17 (20.73)
AG/C1+/AA	44 (41.12)	25 (43.10)	12 (34.29)	41 (50.00)
GG/C1+/AA	30 (28.04)	16 (27.59)	11 (31.43)	24 (29.27)
	N = 295 (%)	N = 170 (%)	N = 97 (%)	N = 190 (%)
AA/C1+/Bx	77 (26.10)	46 (27.06)	23 (23.71)	39 (20.53)
AG/C1+/Bx	157 (53.22)	95 (55.88)	48 (49.48)	101 (53.16)
GG/C1+/Bx	61 (20.68)	**29 (17.06)**[s]	26 (26.80)	50 (26.32)
	N = 87 (%)	N = 48 (%)	N = 29 (%)	N = 52 (%)
AA/C2+/AA	24 (27.59)	14 (29.17)	9 (31.03)	13 (25.00)
AG/C2+/AA	45 (51.72)	25 (52.08)	13 (44.83)	23 (44.23)
GG/C2+/AA	18 (20.69)	9 (18.75)	7 (24.14)	16 (30.77)
	N = 242 (%)	N = 136 (%)	N = 79 (%)	N = 149 (%)
AA/C2+/Bx	64 (26.45)	38 (27.94)	19 (24.05)	30 (20.13)
AG/C2+/Bx	115 (47.52)	67 (49.26)	35 (44.30)	81 (54.36)
GG/C2+/Bx	63 (26.03)	31 (22.79)	25 (31.65)	38 (25.50)
	N = 49 (%)	N = 29 (%)	N = 14 (%)	N = 40 (%)
AA/C1C1/AA	13 (26.53)	7 (24.14)	3 (21.43)	5 (12.50)
AG/C1C1/AA	18 (36.73)	11 (37.93)	5 (35.71)	22 (55.00)
GG/C1C1/AA	18 (36.73)	11 (37.93)	6 (42.86)	13 (32.50)
	N = 58 (%)	N = 29 (%)	N = 21 (%)	N = 42 (%)
AA/C1C2/AA	20 (34.48)	10 (34.48)	9 (42.86)	12 (28.57)
AG/C1C2/AA	26 (44.83)	14 (48.28)	7 (33.33)	19 (45.24)
GG/C1C2/AA	12 (20.69)	5 (17.24)	5 (23.81)	11 (26.19)
	N = 29 (%)	N = 19 (%)	N = 8 (%)	N = 10 (%)
AA/C2C2/AA	4 (13.79)	4 (21.05)	0 (0.00)	1 (10.00)

Male ERAP/female HLA-C/ female KIR	IVF	RIF	SIVF	Fertile
AG/C2C2/AA	19 (65.52)	11 (57.89)	6 (75.00)	4 (40.00)
GG/C2C2/AA	6 (20.69)	4 (21.05)	2 (25.00)	5 (50.00)
	N = 110 (%)	N = 65 (%)	N = 37 (%)	N = 76 (%)
AA/C1C1/Bx	23 (20.91)	14 (21.54)	6 (16.22)	17 (22.37)
AG/C1C1/Bx	**69 (62.73)**[t]	41 (63.08)	23 (62.16)	35 (46.05)
GG/C1C1/Bx	**18 (16.36)**[u]	**10 (15.38)**[w]	8 (21.62)	24 (31.58)
	N = 185 (%)	N = 105 (%)	N = 60 (%)	N = 114 (%)
AA/C1C2/Bx	54 (29.19)	32 (30.48)	17 (28.33)	22 (19.30)
AG/C1C2/Bx	88 (47.57)	54 (51.43)	25 (41.67)	66 (57.89)
GG/C1C2/Bx	43 (23.24)	19 (18.10)	18 (30.00)	26 (22.81)
	N = 57 (%)	N = 31 (%)	N = 19 (%)	N = 35 (%)
AA/C2C2/Bx	10 (17.54)	6 (19.35)	2 (10.53)	8 (22.86)
AG/C2C2/Bx	27 (47.37)	13 (41.94)	10 (52.63)	15 (42.86)
GG/C2C2/Bx	20 (35.09)	12 (38.71)	7 (36.84)	12 (34.29)
ERAP1 rs6861666/HLA-C/KIR	N = 108 (%)	N = 58 (%)	N = 36 (%)	N = 76 (%)
AA/C1+/AA	96 (88.89)	52 (89.66)	31 (86.11)	65 (85.53)
AG/C1+/AA	12 (11.11)	6 (10.34)	5 (13.89)	9 (11.84)
GG/C1+/AA	0 (0.00)	0 (0.00)	0 (0.00)	2 (2.63)
	N = 295 (%)	N = 169 (%)	N = 98 (%)	N = 181 (%)
AA/C1+/Bx	244 (82.71)	146 (86.39)	79 (80.61)	150 (82.87)
AG/C1+/Bx	50 (16.95)	22 (13.02)	19 (19.39)	31 (17.13)
GG/C1+/Bx	1 (0.34)	1 (0.59)	0 (0.00)	0 (0.00)
	N = 88 (%)	N = 48 (%)	N = 30 (%)	N = 46 (%)
AA/C2+/AA	81 (92.05)	44 (91.67)	28 (93.33)	37 (80.43)
AG/C2+/AA	7 (7.95)	4 (8.33)	2 (6.67)	6 (13.04)
GG/C2+/AA	**0 (0.00)**[x]	0 (0.00)	0 (0.00)	3 (6.52)
	N = 243 (%)	N = 137 (%)	N = 79 (%)	N = 139 (%)
AA/C2+/Bx	207 (85.19)	117 (85.40)	68 (86.08)	110 (79.14)
AG/C2+/Bx	35 (14.40)	19 (13.87)	11 (13.92)	29 (20.86)
GG/C2+/Bx	1 (0.41)	1 (0.73)	0 (0.00)	0 (0.00)
	N = 49 (%)	N = 29 (%)	N = 14 (%)	N = 38 (%)
AA/C1C1/AA	41 (83.67)	25 (86.21)	10 (71.43)	33 (86.84)
AG/C1C1/AA	8 (16.33)	4 (13.79)	4 (28.57)	5 (13.16)
GG/C1C1/AA	0 (0.00)	0 (0.00)	0 (0.00)	0 (0.00)
	N = 59 (%)	N = 29 (%)	N = 22 (%)	N = 38 (%)
AA/C1C2/AA	55 (93.22)	27 (93.10)	21 (95.45)	32 (84.21)
AG/C1C2/AA	4 (6.78)	2 (6.90)	1 (4.55)	4 (10.53)
GG/C1C2/AA	0 (0.00)	0 (0.00)	0 (0.00)	2 (5.26)
	N = 29 (%)	N = 19 (%)	N = 8 (%)	N = 8 (%)
AA/C2C2/AA	26 (89.66)	17 (89.47)	7 (87.50)	5 (62.50)
AG/C2C2/AA	3 (10.34)	2 (10.53)	1 (12.50)	2 (25.00)
GG/C2C2/AA	0 (0.00)	0 (0.00)	0 (0.00)	1 (12.50)
	N = 110 (%)	N = 64 (%)	N = 38 (%)	N = 73 (%)
AA/C1C1/Bx	90 (81.82)	57 (89.06)	29 (76.32)	63 (86.30)
AG/C1C1/Bx	20 (18.18)	7 (10.94)	9 (23.68)	10 (13.70)
GG/C1C1/Bx	0 (0.00)	0 (0.00)	0 (0.00)	0 (0.00)

Male ERAP/female HLA-C/ female KIR	IVF	RIF	SIVF	Fertile
	N = 185 (%)	N = 105 (%)	N = 60 (%)	N = 108 (%)
AA/C1C2/Bx	154 (83.24)	89 (84.76)	50 (83.33)	87 (80.56)
AG/C1C2/Bx	30 (16.22)	15 (14.29)	10 (16.67)	21 (19.44)
GG/C1C2/Bx	1 (0.54)	1 (0.95)	0 (0.00)	0 (0.00)
	N = 58 (%)	N = 32 (%)	N = 19 (%)	N = 31 (%)
AA/C2C2/Bx	53 (91.38)	28 (87.50)	18 (94.74)	23 (74.19)
AG/C2C2/Bx	5 (8.62)	4 (12.50)	1 (5.26)	8 (25.81)
GG/C2C2/Bx	0 (0.00)	0 (0.00)	0 (0.00)	0 (0.00)

IVF-ET – in vitro fertilization embryo transfer; RIF – recurrent implantation failure; SIVF – successful pregnancy after IVF-ET; p – probability; $p_{corr.}$ – probability after Bonferroni correction for multiple comparisons (x6 for possible *ERAP* with *HLA-C* C1+ or C2+ and *KIR* AA or Bx combinations; x9 for possible genotypes *ERAP*, *HLA-C* with *KIR* AA or Bx combinations); OR – odds ratio; 95% CI – confidence interval from two-sided Fisher's exact test; ns – not significant. Values in bold indicate significant differences.

IVF *vs.* Fertile: $^{f}p/p_{corr.}$ = 0.049/ns, OR = 2.573, 95% CI (0.96-7.40); $^{k}p/p_{corr.}$ = 0.031/ns, OR = Inf, 95% CI (1.01-Inf); $^{t}p/p_{corr.}$ = 0.035/ns, OR = 1.964, 95% CI (1.04-3.73); $^{u}p/p_{corr.}$ = 0.020/ns, OR = 0.426, 95% CI (0.20-0.90); $^{x}p/p_{corr.}$ = 0.039/ns, OR = 0.000, 95% CI (0.00-1.24);

RIF *vs.* Fertile: $^{a}p/p_{corr.}$ = 0.043/ns, OR = 0.210, 95% CI (0.02-1.00); $^{g}p/p_{corr.}$ = 0.015/ns, OR = 0.114, 95% CI (0.00-0.83); $^{n}p/p_{corr.}$ = 0.041/ns, OR = 3.548, 95% CI (0.93-16.64); $^{s}p/p_{corr.}$ = 0.041/ns, OR = 0.577, 95% CI (0.33-0.99); $^{w}p/p_{corr.}$ = 0.030/ns, OR = 0.397, 95% CI (0.15-0.96);

SIVF *vs.* Fertile: $^{j}p/p_{corr.}$ = 0.052/ns, OR = 2.505, 95% CI (0.90-7.20); $^{q}p/p_{corr.}$ = 0.051/ns, OR = 0.312, 95% CI (0.08-1.12); $^{r}p/p_{corr.}$ = 0.021/ns, OR = 4.160, 95% CI (1.13-16.69)

RIF *vs.* SIVF: $^{b}p/p_{corr.}$ = 0.025/ns, OR = 0.154, 95% CI (0.01-0.88); $^{c}p/p_{corr.}$ = 0.005/0.049, OR = 24.000, 95% CI (1.76-1549.16); $^{d}p/p_{corr.}$ = 0.011/ns, OR = 0.087, 95% CI (0.00-0.77); $^{e}p/p_{corr.}$ = 0.015/ns, OR = 21.399, 95% CI (1.53-1 396.02); $^{h}p/p_{corr.}$ = 0.020/ns, OR = 0.098, 95% CI (0.00-0.92); $^{i}p/p_{corr.}$ = 0.041/ns, OR = 16.199, 95% CI (1.09-1 088.44); $^{l}p/p_{corr.}$ = 0.028/ns, OR = 12.052, 95% CI (1.00-703.94); $^{m}p/p_{corr.}$ = 0.032/ns, OR = 2.105, 95% CI (1.04-4.32); $^{o}p/p_{corr.}$ = 0.010/ns, OR = Inf, 95% CI (1.50-Inf)
