# Supplementary material for: ERAP/HLA-C and KIR Genetic Profile in Couples with Recurrent Implantation Failure
Source: Int J Mol Sci. 2022 Oct 19;23(20):12518. doi: 10.3390/ijms232012518 (PMC9603896; doi:10.3390/ijms232012518)
Supplement: Supplementary file 1 [file ijms-23-12518-s001.zip › Supplementary Table S5.pdf]

Supplementary Table S5. Distribution of female *KIR* with her partner's *ERAP/HLA-C* genotype combination in couples undergoing *in vitro* fertilization and in fertile couples.

Male ERAP/male HLA-C/ female KIR	IVF	RIF	SIVF	Fertile
ERAP1 rs30187/HLA-C/KIR	N = 115 (%)	N = 70 (%)	N = 30 (%)	N = 78 (%)
CC/C1+/AA	57 (49.57)	40 (57.14)	12 (40.00)	38 (48.72)
CT/C1+/AA	47 (40.87)	26 (37.14)	11 (36.67)	29 (37.18)
TT/C1+/AA	11 (9.57)	4 (5.71)[a]	7 (23.33)	11 (14.10)
	N = 304 (%)	N = 166 (%)	N = 106 (%)	N = 190 (%)
CC/C1+/Bx	155 (50.99)	82 (49.40)	58 (54.72)	83 (43.68)
CT/C1+/Bx	119 (39.14)	66 (39.76)	38 (35.85)	86 (45.26)
TT/C1+/Bx	30 (9.87)	18 (10.84)	10 (9.43)	21 (11.05)
	N = 103 (%)	N = 54 (%)	N = 38 (%)	N = 64 (%)
CC/C2+/AA	54 (52.43)	30 (55.56)	19 (50.00)	29 (45.31)
CT/C2+/AA	37 (35.92)	20 (37.04)	11 (28.95)	24 (37.50)
TT/C2+/AA	12 (11.65)	4 (7.41)	8 (21.05)	11 (17.19)
	N = 217 (%)	N = 126 (%)	N = 72 (%)	N = 143 (%)
CC/C2+/Bx	103 (47.47)	60 (47.62)	34 (47.22)	59 (41.26)
CT/C2+/Bx	90 (41.47)	51 (40.48)	31 (43.06)	73 (51.05)
TT/C2+/Bx	24 (11.06)	15 (11.90)	7 (9.72)	11 (7.69)
	N = 35 (%)	N = 23 (%)	N = 6 (%)	N = 28 (%)
CC/C1C1/AA	18 (51.43)	14 (60.87)	2 (33.33)	15 (53.57)
CT/C1C1/AA	14 (40.00)	8 (34.78)	2 (33.33)	11 (39.29)
TT/C1C1/AA	3 (8.57)	1 (4.35)	2 (33.33)	2 (7.14)
	N = 80 (%)	N = 47 (%)	N = 24 (%)	N = 50 (%)
CC/C1C2/AA	39 (48.75)	26 (55.32)	10 (41.67)	23 (46.00)
CT/C1C2/AA	33 (41.25)	18 (38.30)	9 (37.50)	18 (36.00)
TT/C1C2/AA	8 (10.00)	3 (6.38)	5 (20.83)	9 (18.00)
	N = 23 (%)	N = 7 (%)	N = 14 (%)	N = 14 (%)
CC/C2C2/AA	15 (65.22)	4 (57.14)	9 (64.29)	6 (42.86)
CT/C2C2/AA	4 (17.39)	2 (28.57)	2 (14.29)	6 (42.86)
TT/C2C2/AA	4 (17.39)	1 (14.29)	3 (21.43)	2 (14.29)
	N = 136 (%)	N = 75 (%)	N = 45 (%)	N = 83 (%)
CC/C1C1/Bx	73 (53.68)	38 (50.67)	26 (57.78)	41 (49.40)
CT/C1C1/Bx	53 (38.97)	30 (40.00)	16 (35.56)	31 (37.35)
TT/C1C1/Bx	10 (7.35)	7 (9.33)	3 (6.67)	11 (13.25)
	N = 168 (%)	N = 91 (%)	N = 61 (%)	N = 107 (%)
CC/C1C2/Bx	82 (48.81)	44 (48.35)	32 (52.46)	42 (39.25)
CT/C1C2/Bx	66 (39.29)	36 (39.56)	22 (36.07)	55 (51.40)
TT/C1C2/Bx	20 (11.90)	11 (12.09)	7 (11.48)	10 (9.35)
	N = 49 (%)	N = 35 (%)	N = 11 (%)	N = 36 (%)
CC/C2C2/Bx	21 (42.86)	16 (45.71)	2 (18.18)	17 (47.22)
CT/C2C2/Bx	24 (48.98)	15 (42.86)[b]	9 (81.82)	18 (50.00)
TT/C2C2/Bx	4 (8.16)	4 (11.43)	0 (0.00)	1 (2.78)
ERAP1 rs27044/HLA-C/KIR	N = 115 (%)	N = 70 (%)	N = 30 (%)	N = 78 (%)
CC/C1+/AA	66 (57.39)	47 (67.14)	12 (40.00)	46 (58.97)

Male ERAP/male HLA-C/ female KIR	IVF	RIF	SIVF	Fertile
CG/C1+/AA	40 (34.78)	21 (30.00)	11 (36.67)	24 (30.77)
GG/C1+/AA	9 (7.83)	**2 (2.86)**[c]	7 (23.33)	8 (10.26)
	N = 304 (%)	N = 166 (%)	N = 106 (%)	N = 190 (%)
CC/C1+/Bx	180 (59.21)	98 (59.04)	63 (59.43)	102 (53.68)
CG/C1+/Bx	105 (34.54)	60 (36.14)	34 (32.08)	70 (36.84)
GG/C1+/Bx	19 (6.25)	8 (4.82)	9 (8.49)	18 (9.47)
	N = 103 (%)	N = 54 (%)	N = 38 (%)	N = 64 (%)
CC/C2+/AA	60 (58.25)	35 (64.81)	19 (50.00)	34 (53.12)
CG/C2+/AA	33 (32.04)	16 (29.63)	12 (31.58)	23 (35.94)
GG/C2+/AA	10 (9.71)	3 (5.56)	7 (18.42)	7 (10.94)
	N = 217 (%)	N = 126 (%)	N = 72 (%)	N = 143 (%)
CC/C2+/Bx	125 (57.60)	76 (60.32)	37 (51.39)	81 (56.64)
CG/C2+/Bx	77 (35.48)	42 (33.33)	30 (41.67)	52 (36.36)
GG/C2+/Bx	15 (6.91)	8 (6.35)	5 (6.94)	10 (6.99)
	N = 35 (%)	N = 23 (%)	N = 6 (%)	N = 28 (%)
CC/C1C1/AA	21 (60.00)	16 (69.57)	2 (33.33)	19 (67.86)
CG/C1C1/AA	12 (34.29)	7 (30.43)	2 (33.33)	8 (28.57)
GG/C1C1/AA	2 (5.71)	**0 (0.00)**[d]	2 (33.33)	1 (3.57)
	N = 80 (%)	N = 47 (%)	N = 24 (%)	N = 50 (%)
CC/C1C2/AA	45 (56.25)	31 (65.96)	10 (41.67)	27 (54.00)
CG/C1C2/AA	28 (35.00)	14 (29.79)	9 (37.50)	16 (32.00)
GG/C1C2/AA	7 (8.75)	2 (4.26)	5 (20.83)	7 (14.00)
	N = 23 (%)	N = 7 (%)	N = 14 (%)	N = 14 (%)
CC/C2C2/AA	15 (65.22)	4 (57.14)	9 (64.29)	7 (50.00)
CG/C2C2/AA	5 (21.74)	2 (28.57)	3 (21.43)	7 (50.00)
GG/C2C2/AA	3 (13.04)	1 (14.29)	2 (14.29)	0 (0.00)
	N = 136 (%)	N = 75 (%)	N = 45 (%)	N = 83 (%)
CC/C1C1/Bx	81 (59.56)	43 (57.33)	28 (62.22)	43 (51.81)
CG/C1C1/Bx	48 (35.29)	29 (38.67)	13 (28.89)	31 (37.35)
GG/C1C1/Bx	7 (5.15)	3 (4.00)	4 (8.89)	9 (10.84)
	N = 168 (%)	N = 91 (%)	N = 61 (%)	N = 107 (%)
CC/C1C2/Bx	99 (58.93)	55 (60.44)	35 (57.38)	59 (55.14)
CG/C1C2/Bx	57 (33.93)	31 (34.07)	21 (34.43)	39 (36.45)
GG/C1C2/Bx	12 (7.14)	5 (5.49)	5 (8.20)	9 (8.41)
	N = 49 (%)	N = 35 (%)	N = 11 (%)	N = 36 (%)
CC/C2C2/Bx	26 (53.06)	**21 (60.00)**[e]	**2 (18.18)**[f]	22 (61.11)
CG/C2C2/Bx	20 (40.82)	**11 (31.43)**[g]	**9 (81.82)**[h]	13 (36.11)
GG/C2C2/Bx	3 (6.12)	3 (8.57)	0 (0.00)	1 (2.78)
ERAP1 rs26653/HLA-C/KIR	N = 115 (%)	N = 70 (%)	N = 30 (%)	N = 78 (%)
GG/C1+/AA	66 (57.39)	40 (57.14)	17 (56.67)	40 (51.28)
CG/C1+/AA	40 (34.78)	27 (38.57)	8 (26.67)	29 (37.18)
CC/C1+/AA	9 (7.83)	3 (4.29)	5 (16.67)	9 (11.54)
	N = 304 (%)	N = 166 (%)	N = 106 (%)	N = 190 (%)
GG/C1+/Bx	169 (55.59)	93 (56.02)	59 (55.66)	100 (52.63)
CG/C1+/Bx	110 (36.18)	56 (33.73)	40 (37.74)	79 (41.58)
CC/C1+/Bx	25 (8.22)	17 (10.24)	7 (6.60)	11 (5.79)

Male ERAP/male HLA-C/ female KIR	IVF	RIF	SIVF	Fertile
	N = 103 (%)	N = 54 (%)	N = 38 (%)	N = 64 (%)
GG/C2+/AA	62 (60.19)	30 (55.56)	25 (65.79)	32 (50.00)
CG/C2+/AA	34 (33.01)	21 (38.89)	9 (23.68)	22 (34.38)
CC/C2+/AA	7 (6.80)	3 (5.56)	4 (10.53)	10 (15.62)
	N = 217 (%)	N = 126 (%)	N = 72 (%)	N = 143 (%)
GG/C2+/Bx	113 (52.07)	68 (53.97)	37 (51.39)	74 (51.75)
CG/C2+/Bx	81 (37.33)	42 (33.33)	29 (40.28)	60 (41.96)
CC/C2+/Bx	23 (10.60)	16 (12.70)	6 (8.33)	9 (6.29)
	N = 35 (%)	N = 23 (%)	N = 6 (%)	N = 28 (%)
GG/C1C1/AA	20 (57.14)	14 (60.87)	2 (33.33)	16 (57.14)
CG/C1C1/AA	10 (28.57)	8 (34.78)	1 (16.67)	9 (32.14)
CC/C1C1/AA	5 (14.29)	**1 (4.35)**[i]	**3 (50.00)**[j]	3 (10.71)
	N = 80 (%)	N = 47 (%)	N = 24 (%)	N = 50 (%)
GG/C1C2/AA	46 (57.50)	26 (55.32)	15 (62.50)	24 (48.00)
CG/C1C2/AA	30 (37.50)	19 (40.43)	7 (29.17)	20 (40.00)
CC/C1C2/AA	4 (5.00)	2 (4.26)	2 (8.33)	6 (12.00)
	N = 23 (%)	N = 7 (%)	N = 14 (%)	N = 14 (%)
GG/C2C2/AA	16 (69.57)	4 (57.14)	10 (71.43)	8 (57.14)
CG/C2C2/AA	4 (17.39)	2 (28.57)	2 (14.29)	2 (14.29)
CC/C2C2/AA	3 (13.04)	1 (14.29)	2 (14.29)	4 (28.57)
	N = 136 (%)	N = 75 (%)	N = 45 (%)	N = 83 (%)
GG/C1C1/Bx	81 (59.56)	43 (57.33)	28 (62.22)	41 (49.40)
CG/C1C1/Bx	48 (35.29)	27 (36.00)	15 (33.33)	38 (45.78)
CC/C1C1/Bx	7 (5.15)	5 (6.67)	2 (4.44)	4 (4.82)
	N = 168 (%)	N = 91 (%)	N = 61 (%)	N = 107 (%)
GG/C1C2/Bx	88 (52.38)	50 (54.95)	31 (50.82)	59 (55.14)
CG/C1C2/Bx	62 (36.90)	29 (31.87)	25 (40.98)	41 (38.32)
CC/C1C2/Bx	18 (10.71)	12 (13.19)	5 (8.20)	7 (6.54)
	N = 49 (%)	N = 35 (%)	N = 11 (%)	N = 36 (%)
GG/C2C2/Bx	25 (51.02)	18 (51.43)	6 (54.55)	15 (41.67)
CG/C2C2/Bx	19 (38.78)	13 (37.14)	4 (36.36)	19 (52.78)
CC/C2C2/Bx	5 (10.20)	4 (11.43)	1 (9.09)	2 (5.56)
ERAP1 rs26618/HLA-C/KIR	N = 115 (%)	N = 70 (%)	N = 30 (%)	N = 77 (%)
TT/C1+/AA	60 (52.17)	37 (52.86)	18 (60.00)	49 (63.64)
CT/C1+/AA	44 (38.26)	28 (40.00)	8 (26.67)	21 (27.27)
CC/C1+/AA	11 (9.57)	5 (7.14)	4 (13.33)	7 (9.09)
	N = 304 (%)	N = 166 (%)	N = 106 (%)	N = 190 (%)
TT/C1+/Bx	155 (50.99)	87 (52.41)	47 (44.34)	91 (47.89)
CT/C1+/Bx	117 (38.49)	63 (37.95)	46 (43.40)	84 (44.21)
CC/C1+/Bx	32 (10.53)	16 (9.64)	13 (12.26)	15 (7.89)
	N = 103 (%)	N = 54 (%)	N = 38 (%)	N = 63 (%)
TT/C2+/AA	55 (53.40)	29 (53.70)	21 (55.26)	42 (66.67)
CT/C2+/AA	35 (33.98)	18 (33.33)	12 (31.58)	14 (22.22)
CC/C2+/AA	13 (12.62)	7 (12.96)	5 (13.16)	7 (11.11)
	N = 217 (%)	N = 126 (%)	N = 72 (%)	N = 143 (%)
TT/C2+/Bx	114 (52.53)	66 (52.38)	34 (47.22)	69 (48.25)

Male ERAP/male HLA-C/ female KIR	IVF	RIF	SIVF	Fertile
CT/C2+/Bx	85 (39.17)	51 (40.48)	30 (41.67)	65 (45.45)
CC/C2+/Bx	18 (8.29)	9 (7.14)	8 (11.11)	9 (6.29)
	N = 35 (%)	N = 23 (%)	N = 6 (%)	N = 28 (%)
TT/C1C1/AA	18 (51.43)	12 (52.17)	5 (83.33)	16 (57.14)
CT/C1C1/AA	16 (45.71)	11 (47.83)	1 (16.67)	9 (32.14)
CC/C1C1/AA	1 (2.86)	0 (0.00)	0 (0.00)	3 (10.71)
	N = 80 (%)	N = 47 (%)	N = 24 (%)	N = 49 (%)
TT/C1C2/AA	42 (52.50)	25 (53.19)	13 (54.17)	33 (67.35)
CT/C1C2/AA	28 (35.00)	17 (36.17)	7 (29.17)	12 (24.49)
CC/C1C2/AA	10 (12.50)	5 (10.64)	4 (16.67)	4 (8.16)
	N = 23 (%)	N = 7 (%)	N = 14 (%)	N = 14 (%)
TT/C2C2/AA	13 (56.52)	4 (57.14)	8 (57.14)	9 (64.29)
CT/C2C2/AA	7 (30.43)	1 (14.29)	5 (35.71)	2 (14.29)
CC/C2C2/AA	3 (13.04)	2 (28.57)	1 (7.14)	3 (21.43)
	N = 136 (%)	N = 75 (%)	N = 45 (%)	N = 83 (%)
TT/C1C1/Bx	64 (47.06)	37 (49.33)	18 (40.00)	40 (48.19)
CT/C1C1/Bx	55 (40.44)	29 (38.67)	21 (46.67)	36 (43.37)
CC/C1C1/Bx	17 (12.50)	9 (12.00)	6 (13.33)	7 (8.43)
	N = 168 (%)	N = 91 (%)	N = 61 (%)	N = 107 (%)
TT/C1C2/Bx	91 (54.17)	50 (54.95)	29 (47.54)	51 (47.66)
CT/C1C2/Bx	62 (36.90)	34 (37.36)	25 (40.98)	48 (44.86)
CC/C1C2/Bx	15 (8.93)	7 (7.69)	7 (11.48)	8 (7.48)
	N = 49 (%)	N = 35 (%)	N = 11 (%)	N = 36 (%)
TT/C2C2/Bx	23 (46.94)	16 (45.71)	5 (45.45)	18 (50.00)
CT/C2C2/Bx	23 (46.94)	17 (48.57)	5 (45.45)	17 (47.22)
CC/C2C2/Bx	3 (6.12)	2 (5.71)	1 (9.09)	1 (2.78)
ERAP1 rs2287987/HLA-C/KIR	N = 115 (%)	N = 70 (%)	N = 30 (%)	N = 77 (%)
TT/C1+/AA	70 (60.87)	40 (57.14)	20 (66.67)	46 (59.74)
CT/C1+/AA	32 (27.83)	19 (27.14)	8 (26.67)	28 (36.36)
CC/C1+/AA	13 (11.30)	**11 (15.71)**[k]	2 (6.67)	3 (3.90)
	N = 303 (%)	N = 166 (%)	N = 105 (%)	N = 190 (%)
TT/C1+/Bx	188 (62.05)	108 (65.06)	64 (60.95)	122 (64.21)
CT/C1+/Bx	103 (33.99)	50 (30.12)	38 (36.19)	61 (32.11)
CC/C1+/Bx	12 (3.96)	8 (4.82)	3 (2.86)	7 (3.68)
	N = 103 (%)	N = 54 (%)	N = 38 (%)	N = 63 (%)
TT/C2+/AA	60 (58.25)	32 (59.26)	23 (60.53)	40 (63.49)
CT/C2+/AA	31 (30.10)	15 (27.78)	11 (28.95)	21 (33.33)
CC/C2+/AA	12 (11.65)	7 (12.96)	4 (10.53)	2 (3.17)
	N = 217 (%)	N = 126 (%)	N = 72 (%)	N = 143 (%)
TT/C2+/Bx	146 (67.28)	85 (67.46)	49 (68.06)	88 (61.54)
CT/C2+/Bx	63 (29.03)	36 (28.57)	21 (29.17)	50 (34.97)
CC/C2+/Bx	8 (3.69)	5 (3.97)	2 (2.78)	5 (3.50)
	N = 35 (%)	N = 23 (%)	N = 6 (%)	N = 28 (%)
TT/C1C1/AA	23 (65.71)	13 (56.52)	5 (83.33)	17 (60.71)
CT/C1C1/AA	8 (22.86)	6 (26.09)	1 (16.67)	9 (32.14)
CC/C1C1/AA	4 (11.43)	4 (17.39)	0 (0.00)	2 (7.14)

Male ERAP/male HLA-C/ female KIR	IVF	RIF	SIVF	Fertile
	N = 80 (%)	N = 47 (%)	N = 24 (%)	N = 49 (%)
TT/C1C2/AA	47 (58.75)	27 (57.45)	15 (62.50)	29 (59.18)
CT/C1C2/AA	24 (30.00)	13 (27.66)	7 (29.17)	19 (38.78)
CC/C1C2/AA	9 (11.25)	**7 (14.89)**[l]	2 (8.33)	1 (2.04)
	N = 23 (%)	N = 7 (%)	N = 14 (%)	N = 14 (%)
TT/C2C2/AA	13 (56.52)	5 (71.43)	8 (57.14)	11 (78.57)
CT/C2C2/AA	7 (30.43)	2 (28.57)	4 (28.57)	2 (14.29)
CC/C2C2/AA	3 (13.04)	0 (0.00)	2 (14.29)	1 (7.14)
	N = 135 (%)	N = 75 (%)	N = 44 (%)	N = 83 (%)
TT/C1C1/Bx	75 (55.56)	46 (61.33)	23 (52.27)	53 (63.86)
CT/C1C1/Bx	53 (39.26)	24 (32.00)	20 (45.45)	28 (33.73)
CC/C1C1/Bx	7 (5.19)	5 (6.67)	1 (2.27)	2 (2.41)
	N = 168 (%)	N = 91 (%)	N = 61 (%)	N = 107 (%)
TT/C1C2/Bx	113 (67.26)	62 (68.13)	41 (67.21)	69 (64.49)
CT/C1C2/Bx	50 (29.76)	26 (28.57)	18 (29.51)	33 (30.84)
CC/C1C2/Bx	5 (2.98)	3 (3.30)	2 (3.28)	5 (4.67)
	N = 49 (%)	N = 35 (%)	N = 11 (%)	N = 36 (%)
TT/C2C2/Bx	33 (67.35)	23 (65.71)	8 (72.73)	19 (52.78)
CT/C2C2/Bx	13 (26.53)	10 (28.57)	3 (27.27)	17 (47.22)
CC/C2C2/Bx	3 (6.12)	2 (5.71)	0 (0.00)	0 (0.00)
ERAP2 rs2248374/HLA-C/KIR	N = 114 (%)	N = 70 (%)	N = 29 (%)	N = 78 (%)
AA/C1+/AA	**32 (28.07)**[m]	19 (27.14)	8 (27.59)	12 (15.38)
AG/C1+/AA	52 (45.61)	33 (47.14)	11 (37.93)	40 (51.28)
GG/C1+/AA	30 (26.32)	18 (25.71)	10 (34.48)	26 (33.33)
	N = 303 (%)	N = 166 (%)	N = 105 (%)	N = 190 (%)
AA/C1+/Bx	74 (24.42)	43 (25.90)	22 (20.95)	42 (22.11)
AG/C1+/Bx	159 (52.48)	89 (53.61)	53 (50.48)	97 (51.05)
GG/C1+/Bx	70 (23.10)	34 (20.48)	30 (28.57)	51 (26.84)
	N = 102 (%)	N = 54 (%)	N = 37 (%)	N = 64 (%)
AA/C2+/AA	29 (28.43)	14 (25.93)	11 (29.73)	13 (20.31)
AG/C2+/AA	47 (46.08)	27 (50.00)	16 (43.24)	26 (40.62)
GG/C2+/AA	26 (25.49)	13 (24.07)	10 (27.03)	25 (39.06)
	N = 216 (%)	N = 126 (%)	N = 71 (%)	N = 143 (%)
AA/C2+/Bx	55 (25.46)	34 (26.98)	15 (21.13)	26 (18.18)
AG/C2+/Bx	105 (48.61)	63 (50.00)	35 (49.30)	75 (52.45)
GG/C2+/Bx	56 (25.93)	29 (23.02)	21 (29.58)	42 (29.37)
	N = 35 (%)	N = 23 (%)	N = 6 (%)	N = 28 (%)
AA/C1C1/AA	9 (25.71)	7 (30.43)	1 (16.67)	5 (17.86)
AG/C1C1/AA	16 (45.71)	**9 (39.13)**[n]	2 (33.33)	19 (67.86)
GG/C1C1/AA	10 (28.57)	7 (30.43)	3 (50.00)	4 (14.29)
	N = 79 (%)	N = 47 (%)	N = 23 (%)	N = 50 (%)
AA/C1C2/AA	23 (29.11)	12 (25.53)	7 (30.43)	7 (14.00)
AG/C1C2/AA	36 (45.57)	24 (51.06)	9 (39.13)	21 (42.00)
GG/C1C2/AA	**20 (25.32)**[o]	**11 (23.40)**[p]	7 (30.43)	22 (44.00)
	N = 23 (%)	N = 7 (%)	N = 14 (%)	N = 14 (%)
AA/C2C2/AA	6 (26.09)	2 (28.57)	4 (28.57)	6 (42.86)

Male ERAP/male HLA-C/ female KIR	IVF	RIF	SIVF	Fertile
AG/C2C2/AA	11 (47.83)	3 (42.86)	7 (50.00)	5 (35.71)
GG/C2C2/AA	6 (26.09)	2 (28.57)	3 (21.43)	3 (21.43)
	N = 136 (%)	N = 75 (%)	N = 45 (%)	N = 83 (%)
AA/C1C1/Bx	32 (23.53)	18 (24.00)	10 (22.22)	21 (25.30)
AG/C1C1/Bx	79 (58.09)	45 (60.00)	23 (51.11)	41 (49.40)
GG/C1C1/Bx	25 (18.38)	12 (16.00)	12 (26.67)	21 (25.30)
	N = 167 (%)	N = 91 (%)	N = 60 (%)	N = 107 (%)
AA/C1C2/Bx	42 (25.15)	25 (27.47)	12 (20.00)	21 (19.63)
AG/C1C2/Bx	80 (47.90)	44 (48.35)	30 (50.00)	56 (52.34)
GG/C1C2/Bx	45 (26.95)	22 (24.18)	18 (30.00)	30 (28.04)
	N = 49 (%)	N = 35 (%)	N = 11 (%)	N = 36 (%)
AA/C2C2/Bx	13 (26.53)	9 (25.71)	3 (27.27)	5 (13.89)
AG/C2C2/Bx	25 (51.02)	19 (54.29)	5 (45.45)	19 (52.78)
GG/C2C2/Bx	11 (22.45)	7 (20.00)	3 (27.27)	12 (33.33)
ERAP1 rs6861666/HLA-C/KIR	N = 115 (%)	N = 70 (%)	N = 30 (%)	N = 73 (%)
AA/C1+/AA	102 (88.70)	62 (88.57)	26 (86.67)	61 (83.56)
AG/C1+/AA	13 (11.30)	8 (11.43)	4 (13.33)	9 (12.33)
GG/C1+/AA	0 (0.00)	0 (0.00)	0 (0.00)	3 (4.11)
	N = 302 (%)	N = 164 (%)	N = 106 (%)	N = 178 (%)
AA/C1+/Bx	255 (84.44)	142 (86.59)	89 (83.96)	145 (81.46)
AG/C1+/Bx	46 (15.23)	21 (12.80)	17 (16.04)	33 (18.54)
GG/C1+/Bx	1 (0.33)	1 (0.61)	0 (0.00)	0 (0.00)
	N = 103 (%)	N = 54 (%)	N = 38 (%)	N = 57 (%)
AA/C2+/AA	92 (89.32)	49 (90.74)	32 (84.21)	47 (82.46)
AG/C2+/AA	11 (10.68)	5 (9.26)	6 (15.79)	8 (14.04)
GG/C2+/AA	0 (0.00)	0 (0.00)	0 (0.00)	2 (3.51)
	N = 216 (%)	N = 125 (%)	N = 72 (%)	N = 134 (%)
AA/C2+/Bx	179 (82.87)	109 (87.20)	57 (79.17)	114 (85.07)
AG/C2+/Bx	36 (16.67)	15 (12.00)	15 (20.83)	20 (14.93)
GG/C2+/Bx	1 (0.46)	1 (0.80)	0 (0.00)	0 (0.00)
	N = 35 (%)	N = 23 (%)	N = 6 (%)	N = 27 (%)
AA/C1C1/AA	31 (88.57)	20 (86.96)	6 (100.00)	23 (85.19)
AG/C1C1/AA	4 (11.43)	3 (13.04)	0 (0.00)	3 (11.11)
GG/C1C1/AA	0 (0.00)	0 (0.00)	0 (0.00)	1 (3.70)
	N = 80 (%)	N = 47 (%)	N = 24 (%)	N = 46 (%)
AA/C1C2/AA	71 (88.75)	42 (89.36)	20 (83.33)	38 (82.61)
AG/C1C2/AA	9 (11.25)	5 (10.64)	4 (16.67)	6 (13.04)
GG/C1C2/AA	0 (0.00)	0 (0.00)	0 (0.00)	2 (4.35)
	N = 23 (%)	N = 7 (%)	N = 14 (%)	N = 11 (%)
AA/C2C2/AA	21 (91.30)	7 (100.00)	12 (85.71)	9 (81.82)
AG/C2C2/AA	2 (8.70)	0 (0.00)	2 (14.29)	2 (18.18)
GG/C2C2/AA	0 (0.00)	0 (0.00)	0 (0.00)	0 (0.00)
	N = 135 (%)	N = 74 (%)	N = 45 (%)	N = 78 (%)
AA/C1C1/Bx	116 (85.93)	63 (85.14)	40 (88.89)	59 (75.64)
AG/C1C1/Bx	19 (14.07)	11 (14.86)	5 (11.11)	19 (24.36)
GG/C1C1/Bx	0 (0.00)	0 (0.00)	0 (0.00)	0 (0.00)

Male ERAP/male HLA-C/ female KIR	IVF	RIF	SIVF	Fertile
	N = 167 (%)	N = 90 (%)	N = 61 (%)	N = 100 (%)
AA/C1C2/Bx	139 (83.23)	79 (87.78)	49 (80.33)	86 (86.00)
AG/C1C2/Bx	27 (16.17)	10 (11.11)	12 (19.67)	14 (14.00)
GG/C1C2/Bx	1 (0.60)	1 (1.11)	0 (0.00)	0 (0.00)
	N = 49 (%)	N = 35 (%)	N = 11 (%)	N = 34 (%)
AA/C2C2/Bx	40 (81.63)	30 (85.71)	8 (72.73)	28 (82.35)
AG/C2C2/Bx	9 (18.37)	5 (14.29)	3 (27.27)	6 (17.65)
GG/C2C2/Bx	0 (0.00)	0 (0.00)	0 (0.00)	0 (0.00)

IVF-ET – in vitro fertilization embryo transfer; RIF – recurrent implantation failure; SIVF – successful pregnancy after IVF-ET; p – probability; $p_{corr.}$ – probability after Bonferroni correction for multiple comparisons (x6 for possible *ERAP* with *HLA-C* C1+ or C2+ and *KIR* AA or Bx combinations; x9 for possible genotypes *ERAP*, *HLA-C* with *KIR* AA or Bx combinations); OR – odds ratio; 95% CI – confidence interval from two-sided Fisher's exact test; ns – not significant. Values in bold indicate significant differences.

IVF *vs.* Fertile: $^{m}p/p_{corr.}$ = 0.054/ns, OR = 2.138, 95% CI (0.98-4.93); $^{o}p/p_{corr.}$ = 0.034/ns, OR = 0.434, 95% CI (0.19-0.98)

RIF *vs.* Fertile: $^{k}p/p_{corr.}$ = 0.022/ns, OR = 4.554, 95% CI (1.13-26.58); $^{l}p/p_{corr.}$ = 0.029/ns, OR = 8.246, 95% CI (0.99-385.54); $^{n}p/p_{corr.}$ = 0.052/ns, OR = 0.312, 95% CI (0.08-1.11); $^{p}p/p_{corr.}$ = 0.053/ns, OR = 0.393, 95% CI (0.15-1.01)

SIVF *vs.* Fertile: $^{f}p/p_{corr.}$ = 0.017/ns, OR = 0.147, 95% CI (0.01-0.86); $^{h}p/p_{corr.}$ = 0.014/ns, OR = 7.606, 95% CI (1.30-82.62); $^{j}p/p_{corr.}$ = 0.053/ns, OR = 7.582. 95% CI (0.70-91.51)

RIF *vs.* SIVF: $^{a}p/p_{corr.}$ = 0.039/ns, OR = 0.243, 95% CI (0.05-1.07); $^{b}p/p_{corr.}$ = 0.015/ns, OR = 0.129, 95% CI (0.01-0.73); $^{c}p/p_{corr.}$ = 0.005/0.031, OR = 0.114, 95% CI (0.01-0.66); $^{d}p/p_{corr.}$ = 0.046/ns, OR = 0.000, 95% CI (0.00-1.47); $^{e}p/p_{corr.}$ = 0.014/ns, OR = 7.501, 95% CI (1.26-82.40); $^{g}p/p_{corr.}$ = 0.004/0.033, OR = 0.096, 95% CI (0.01-0.56); $^{i}p/p_{corr.}$ = 0.022/ns, OR = 0.057, 95% CI (0.00-0.95)
