# Supplementary material for: ERAP/HLA-C and KIR Genetic Profile in Couples with Recurrent Implantation Failure
Source: Int J Mol Sci. 2022 Oct 19;23(20):12518. doi: 10.3390/ijms232012518 (PMC9603896; doi:10.3390/ijms232012518)
Supplement: Supplementary file 1 [file ijms-23-12518-s001.zip › Supplementary Table S6.pdf]

Supplementary Table S6. Distribution of female *HLA-C/ERAP* with her partner's *HLA-C* genotype combinations in couples undergoing *in vitro* fertilization and in fertile couples.

Female HLA-C/female ERAP/male HLA-C	IVF	RIF	SIVF	Fertile
HLA-C/ERAP1 rs30187/HLA-C	N = 343 (%)	N = 193 (%)	N = 113 (%)	N = 234 (%)
C1+/CC/C1+	154 (44.90)	80 (41.45)	53 (46.90)	109 (46.58)
C1+/CT/C1+	152 (44.31)	92 (47.67)	47 (41.59)	105 (44.87)
C1+/TT/C1+	37 (10.79)	21 (10.88)	13 (11.50)	20 (8.55)
	N = 260 (%)	N = 141 (%)	N = 91 (%)	N = 179 (%)
C1+/CC/C2+	118 (45.38)	61 (43.26)	43 (47.25)	91 (50.84)
C1+/CT/C2+	112 (43.08)	62 (43.97)	39 (42.86)	76 (42.46)
C1+/TT/C2+	30 (11.54)	18 (12.77)	9 (9.89)	12 (6.70)
	N = 285 (%)	N = 157 (%)	N = 95 (%)	N = 170 (%)
C2+/CC/C1+	126 (44.21)	61 (38.85)	46 (48.42)	80 (47.06)
C2+/CT/C1+	128 (44.91)	74 (47.13)	42 (44.21)	81 (47.65)
C2+/TT/C1+	31 (10.88)	**22 (14.01)**[a]	7 (7.37)	9 (5.29)
	N = 210 (%)	N = 121 (%)	N = 71 (%)	N = 132 (%)
C2+/CC/C2+	95 (45.24)	51 (42.15)	34 (47.89)	66 (50.00)
C2+/CT/C2+	89 (42.38)	51 (42.15)	32 (45.07)	57 (43.18)
C2+/TT/C2+	26 (12.38)	**19 (15.70)**[b]	5 (7.04)	9 (6.82)
HLA-C/ERAP1 rs27044/HLA-C	N = 343 (%)	N = 193 (%)	N = 113 (%)	N = 234 (%)
C1+/CC/C1+	184 (53.64)	97 (50.26)	61 (53.98)	139 (59.40)
C1+/CG/C1+	135 (39.36)	82 (42.49)	44 (38.94)	78 (33.33)
C1+/GG/C1+	24 (7.00)	14 (7.25)	8 (7.08)	17 (7.26)
	N = 260 (%)	N = 141 (%)	N = 91 (%)	N = 179 (%)
C1+/CC/C2+	140 (53.85)	**71 (50.35)**[c]	49 (53.85)	113 (63.13)
C1+/CG/C2+	101 (38.85)	57 (40.43)	37 (40.66)	54 (30.17)
C1+/GG/C2+	19 (7.31)	13 (9.22)	5 (5.49)	12 (6.70)
	N = 285 (%)	N = 157 (%)	N = 95 (%)	N = 170 (%)
C2+/CC/C1+	155 (54.39)	78 (49.68)	53 (55.79)	99 (58.24)
C2+/CG/C1+	112 (39.30)	68 (43.31)	37 (38.95)	65 (38.24)
C2+/GG/C1+	18 (6.32)	11 (7.01)	5 (5.26)	6 (3.53)
	N = 210 (%)	N = 121 (%)	N = 71 (%)	N = 132 (%)
C2+/CC/C2+	113 (53.81)	61 (50.41)	37 (52.11)	78 (59.09)
C2+/CG/C2+	83 (39.52)	49 (40.50)	32 (45.07)	47 (35.61)
C2+/GG/C2+	14 (6.67)	11 (9.09)	2 (2.82)	7 (5.30)
HLA-C/ERAP1 rs26653/HLA-C	N = 343 (%)	N = 193 (%)	N = 113 (%)	N = 232 (%)
C1+/GG/C1+	195 (56.85)	107 (55.44)	64 (56.64)	121 (52.16)
C1+/CG/C1+	132 (38.48)	78 (40.41)	45 (39.82)	97 (41.81)
C1+/CC/C1+	16 (4.66)	8 (4.15)	4 (3.54)	14 (6.03)
	N = 260 (%)	N = 141 (%)	N = 91 (%)	N = 178 (%)
C1+/GG/C2+	151 (58.08)	80 (56.74)	54 (59.34)	99 (55.62)
C1+/CG/C2+	94 (36.15)	53 (37.59)	35 (38.46)	71 (39.89)
C1+/CC/C2+	15 (5.77)	8 (5.67)	2 (2.20)	8 (4.49)
	N = 285 (%)	N = 157 (%)	N = 95 (%)	N = 168 (%)
C2+/GG/C1+	158 (55.44)	81 (51.59)	57 (60.00)	95 (56.55)
C2+/CG/C1+	116 (40.70)	70 (44.59)	34 (35.79)	66 (39.29)
C2+/CC/C1+	11 (3.86)	6 (3.82)	4 (4.21)	7 (4.17)

Female HLA-C/female ERAP/male HLA-C	IVF	RIF	SIVF	Fertile
	N = 210 (%)	N = 121 (%)	N = 71 (%)	N = 131 (%)
C2+/GG/C2+	119 (56.67)	66 (54.55)	43 (60.56)	77 (58.78)
C2+/CG/C2+	81 (38.57)	50 (41.32)	25 (35.21)	48 (36.64)
C2+/CC/C2+	10 (4.76)	5 (4.13)	3 (4.23)	6 (4.58)
HLA-C/ERAP1 rs26618/HLA-C	N = 343 (%)	N = 193 (%)	N = 113 (%)	N = 234 (%)
C1+/TT/C1+	174 (50.73)	103 (53.37)	55 (48.67)	112 (47.86)
C1+/CT/C1+	143 (41.69)	76 (39.38)	47 (41.59)	103 (44.02)
C1+/CC/C1+	26 (7.58)	14 (7.25)	11 (9.73)	19 (8.12)
	N = 260 (%)	N = 141 (%)	N = 91 (%)	N = 179 (%)
C1+/TT/C2+	136 (52.31)	82 (58.16)	42 (46.15)	86 (48.04)
C1+/CT/C2+	103 (39.62)	48 (34.04)	41 (45.05)	76 (42.46)
C1+/CC/C2+	21 (8.08)	11 (7.80)	8 (8.79)	17 (9.50)
	N = 285 (%)	N = 157 (%)	N = 95 (%)	N = 170 (%)
C2+/TT/C1+	156 (54.74)	95 (60.51)[d]	46 (48.42)	79 (46.47)
C2+/CT/C1+	109 (38.25)	55 (35.03)	39 (41.05)	78 (45.88)
C2+/CC/C1+	20 (7.02)	7 (4.46)	10 (10.53)	13 (7.65)
	N = 210 (%)	N = 121 (%)	N = 71 (%)	N = 132 (%)
C2+/TT/C2+	113 (53.81)	74 (61.16)[e.f]	31 (43.66)	62 (46.97)
C2+/CT/C2+	80 (38.10)	41 (33.88)	31 (43.66)	59 (44.70)
C2+/CC/C2+	17 (8.10)	6 (4.96)	9 (12.68)	11 (8.33)
HLA-C/ERAP1 rs2287987/HLA-C	N = 343 (%)	N = 193 (%)	N = 113 (%)	N = 234 (%)
C1+/TT/C1+	209 (60.93)	116 (60.10)	75 (66.37)	146 (62.39)
C1+/CT/C1+	117 (34.11)	69 (35.75)	31 (27.43)	82 (35.04)
C1+/CC/C1+	17 (4.96)	8 (4.15)	7 (6.19)	6 (2.56)
	N = 260 (%)	N = 141 (%)	N = 91 (%)	N = 179 (%)
C1+/TT/C2+	160 (61.54)	86 (60.99)	58 (63.74)	105 (58.66)
C1+/CT/C2+	84 (32.31)	47 (33.33)	27 (29.67)	68 (37.99)
C1+/CC/C2+	16 (6.15)	8 (5.67)	6 (6.59)	6 (3.35)
	N = 285 (%)	N = 157 (%)	N = 95 (%)	N = 170 (%)
C2+/TT/C1+	174 (61.05)	93 (59.24)	62 (65.26)	109 (64.12)
C2+/CT/C1+	95 (33.33)	56 (35.67)	27 (28.42)	55 (32.35)
C2+/CC/C1+	16 (5.61)	8 (5.10)	6 (6.32)	6 (3.53)
	N = 210 (%)	N = 121 (%)	N = 71 (%)	N = 132 (%)
C2+/TT/C2+	132 (62.86)	75 (61.98)	46 (64.79)	82 (62.12)
C2+/CT/C2+	65 (30.95)	39 (32.23)	21 (29.58)	46 (34.85)
C2+/CC/C2+	13 (6.19)	7 (5.79)	4 (5.63)	4 (3.03)
HLA-C/ERAP2 rs2248374 /HLA-C	N = 342 (%)	N = 192 (%)	N = 113 (%)	N = 232 (%)
C1+/AA/C1+	96 (28.07)	52 (27.08)	37 (32.74)	65 (28.02)
C1+/AG/C1+	163 (47.66)	91 (47.40)	52 (46.02)	116 (50.00)
C1+/GG/C1+	83 (24.27)	49 (25.52)	24 (21.24)	51 (21.98)
	N = 259 (%)	N = 140 (%)	N = 91 (%)	N = 178 (%)
C1+/AA/C2+	73 (28.19)	36 (25.71)	33 (36.26)	48 (26.97)
C1+/AG/C2+	127 (49.03)	66 (47.14)	43 (47.25)	88 (49.44)
C1+/GG/C2+	59 (22.78)	38 (27.14)	15 (16.48)	42 (23.60)
	N = 284 (%)	N = 156 (%)	N = 95 (%)	N = 168 (%)
C2+/AA/C1+	67 (23.59)	36 (23.08)	26 (27.37)	45 (26.79)
C2+/AG/C1+	140 (49.30)	77 (49.36)	44 (46.32)	83 (49.40)

Female HLA-C/female ERAP/male HLA-C	IVF	RIF	SIVF	Fertile
C2+/GG/C1+	77 (27.11)	43 (27.56)	25 (26.32)	40 (23.81)
	N = 209 (%)	N = 120 (%)	N = 71 (%)	N = 131 (%)
C2+/AA/C2+	53 (25.36)	26 (21.67)	24 (33.80)	30 (22.90)
C2+/AG/C2+	102 (48.80)	59 (49.17)	31 (43.66)	64 (48.85)
C2+/GG/C2+	54 (25.84)	35 (29.17)	16 (22.54)	37 (28.24)
HLA-C/ERAP1 rs6861666/HLA-C	N = 342 (%)	N = 192 (%)	N = 113 (%)	N = 231 (%)
C1+/AA/C1+	294 (85.96)	161 (83.85)	98 (86.73)	202 (87.45)
C1+/AG/C1+	47 (13.74)	30 (15.62)	15 (13.27)	29 (12.55)
C1+/GG/C1+	1 (0.29)	1 (0.52)	0 (0.00)	0 (0.00)
	N = 259 (%)	N = 140 (%)	N = 91 (%)	N = 177 (%)
C1+/AA/C2+	219 (84.56)	118 (84.29)	76 (83.52)	153 (86.44)
C1+/AG/C2+	40 (15.44)	22 (15.71)	15 (16.48)	24 (13.56)
C1+/GG/C2+	0 (0.00)	0 (0.00)	0 (0.00)	0 (0.00)
	N = 284 (%)	N = 156 (%)	N = 95 (%)	N = 166 (%)
C2+/AA/C1+	248 (87.32)	134 (85.90)	82 (86.32)	141 (84.94)
C2+/AG/C1+	34 (11.97)	21 (13.46)	12 (12.63)	25 (15.06)
C2+/GG/C1+	2 (0.70)	1 (0.64)	1 (1.05)	0 (0.00)
	N = 209 (%)	N = 120 (%)	N = 71 (%)	N = 130 (%)
C2+/AA/C2+	185 (88.52)	107 (89.17)	62 (87.32)	106 (81.54)
C2+/AG/C2+	24 (11.48)	13 (10.83)	9 (12.68)	23 (17.69)
C2+/GG/C2+	0 (0.00)	0 (0.00)	0 (0.00)	1 (0.77)

IVF-ET – in vitro fertilization embryo transfer; RIF – recurrent implantation failure; SIVF – successful pregnancy after IVF-ET; p – probability; $p_{corr.}$ – probability after Bonferroni correction for multiple comparisons (x12 for possible *HLA-C* alleles); OR – odds ratio; 95% CI – confidence interval from two-sided Fisher's exact test; ns – not significant. Values in bold indicate significant differences.

RIF *vs.* fertile: $^{a}p/p_{corr.}$ = 0.008/ns, OR = 2.906, 95% CI (1.24-7.42); $^{b}p/p_{corr.}$ = 0.028/ns, OR = 2.537, 95% CI (1.04-6.66); $^{c}p/p_{corr.}$ = 0.023/ns, OR = 0.593, 95% CI (0.37-0.95); $^{d}p/p_{corr.}$ = 0.015/ns, OR = 1.762, 95% CI (1.11-2.81); $^{e}p/p_{corr.}$ = 0.032/ns, OR = 1.774, 95% CI (1.05-3.03)

RIF *vs.* SIVF: $^{f}p/p_{corr.}$ = 0.024/ns, OR = 2.024, 95% CI (1.07-3.85)
