# Supplementary material for: ERAP/HLA-C and KIR Genetic Profile in Couples with Recurrent Implantation Failure
Source: Int J Mol Sci. 2022 Oct 19;23(20):12518. doi: 10.3390/ijms232012518 (PMC9603896; doi:10.3390/ijms232012518)
Supplement: Supplementary file 1 [file ijms-23-12518-s001.zip › Supplementary Table S7.pdf]

Supplementary Table S7. Distribution of female *HLA-C* with male *ERAP/HLA-C* genotype combinations in couples undergoing *in vitro* fertilization and in fertile couples.

Female HLA-C/male ERAP/male HLA-C	IVF	RIF	SIVF	Fertile
HLA-C/ERAP1 rs30187/HLA-C	N = 343 (%)	N = 193 (%)	N = 113 (%)	N = 231 (%)
C1+/CC/C1+	181 (52.77)	104 (53.89)	59 (52.21)	105 (45.45)
C1+/CT/C1+	128 (37.32)	72 (37.31)	39 (34.51)	99 (42.86)
C1+/TT/C1+	34 (9.91)	17 (8.81)	15 (13.27)	27 (11.69)
	N = 260 (%)	N = 141 (%)	N = 91 (%)	N = 177 (%)
C1+/CC/C2+	**131 (50.38)**[a]	**74 (52.48)**[b]	43 (47.25)	72 (40.68)
C1+/CT/C2+	100 (38.46)	53 (37.59)	35 (38.46)	84 (47.46)
C1+/TT/C2+	29 (11.15)	14 (9.93)	13 (14.29)	21 (11.86)
	N = 285 (%)	N = 157 (%)	N = 95 (%)	N = 166 (%)
C2+/CC/C1+	135 (47.37)	77 (49.04)	46 (48.42)	72 (43.37)
C2+/CT/C1+	120 (42.11)	62 (39.49)	38 (40.00)	75 (45.18)
C2+/TT/C1+	30 (10.53)	18 (11.46)	11 (11.58)	19 (11.45)
	N = 210 (%)	N = 121 (%)	N = 71 (%)	N = 129 (%)
C2+/CC/C2+	95 (45.24)	56 (46.28)	32 (45.07)	49 (37.98)
C2+/CT/C2+	91 (43.33)	50 (41.32)	31 (43.66)	68 (52.71)
C2+/TT/C2+	24 (11.43)	15 (12.40)	8 (11.27)	12 (9.30)
HLA-C/ERAP1 rs27044/HLA-C	N = 343 (%)	N = 193 (%)	N = 113 (%)	N = 231 (%)
C1+/CC/C1+	209 (60.93)	123 (63.73)	63 (55.75)	128 (55.41)
C1+/CG/C1+	110 (32.07)	62 (32.12)	36 (31.86)	83 (35.93)
C1+/GG/C1+	24 (7.00)	**8 (4.15)**[c]	14 (12.39)	20 (8.66)
	N = 260 (%)	N = 141 (%)	N = 91 (%)	N = 177 (%)
C1+/CC/C2+	155 (59.62)	**92 (65.25)**[d,e]	45 (49.45)	94 (53.11)
C1+/CG/C2+	85 (32.69)	41 (29.08)	36 (39.56)	68 (38.42)
C1+/GG/C2+	20 (7.69)	8 (5.67)	10 (10.99)	15 (8.47)
	N = 285 (%)	N = 157 (%)	N = 95 (%)	N = 166 (%)
C2+/CC/C1+	159 (55.79)	92 (58.60)	50 (52.63)	87 (52.41)
C2+/CG/C1+	104 (36.49)	56 (35.67)	33 (34.74)	63 (37.95)
C2+/GG/C1+	22 (7.72)	9 (5.73)	12 (12.63)	16 (9.64)
	N = 210 (%)	N = 121 (%)	N = 71 (%)	N = 129 (%)
C2+/CC/C2+	112 (53.33)	67 (55.37)	35 (49.30)	65 (50.39)
C2+/CG/C2+	79 (37.62)	44 (36.36)	28 (39.44)	54 (41.86)
C2+/GG/C2+	19 (9.05)	10 (8.26)	8 (11.27)	10 (7.75)
HLA-C/ERAP1 rs26653/HLA-C	N = 343 (%)	N = 193 (%)	N = 113 (%)	N = 231 (%)
C1+/GG/C1+	191 (55.69)	108 (55.96)	62 (54.87)	120 (51.95)
C1+/CG/C1+	127 (37.03)	71 (36.79)	42 (37.17)	97 (41.99)
C1+/CC/C1+	25 (7.29)	14 (7.25)	9 (7.96)	14 (6.06)
	N = 260 (%)	N = 141 (%)	N = 91 (%)	N = 177 (%)
C1+/GG/C2+	141 (54.23)	78 (55.32)	48 (52.75)	89 (50.28)
C1+/CG/C2+	97 (37.31)	51 (36.17)	34 (37.36)	72 (40.68)
C1+/CC/C2+	22 (8.46)	12 (8.51)	9 (9.89)	16 (9.04)
	N = 285 (%)	N = 157 (%)	N = 95 (%)	N = 166 (%)
C2+/GG/C1+	161 (56.49)	90 (57.32)	53 (55.79)	89 (53.61)
C2+/CG/C1+	98 (34.39)	51 (32.48)	34 (35.79)	63 (37.95)
C2+/CC/C1+	26 (9.12)	16 (10.19)	8 (8.42)	14 (8.43)

Female HLA-C/male ERAP/male HLA-C	IVF	RIF	SIVF	Fertile
	N = 210 (%)	N = 121 (%)	N = 71 (%)	N = 129 (%)
C2+/GG/C2+	115 (54.76)	65 (53.72)	41 (57.75)	65 (50.39)
C2+/CG/C2+	75 (35.71)	43 (35.54)	24 (33.80)	50 (38.76)
C2+/CC/C2+	20 (9.52)	13 (10.74)	6 (8.45)	14 (10.85)
HLA-C/ERAP1 rs26618/HLA-C	N = 343 (%)	N = 193 (%)	N = 113 (%)	N = 231 (%)
C1+/TT/C1+	171 (49.85)	102 (52.85)	49 (43.36)	118 (51.08)
C1+/CT/C1+	136 (39.65)	76 (39.38)	48 (42.48)	94 (40.69)
C1+/CC/C1+	36 (10.50)	15 (7.77)	16 (14.16)	19 (8.23)
	N = 260 (%)	N = 141 (%)	N = 91 (%)	N = 177 (%)
C1+/TT/C2+	135 (51.92)	74 (52.48)	43 (47.25)	93 (52.54)
C1+/CT/C2+	100 (38.46)	55 (39.01)	37 (40.66)	69 (38.98)
C1+/CC/C2+	25 (9.62)	12 (8.51)	11 (12.09)	15 (8.47)
	N = 285 (%)	N = 157 (%)	N = 95 (%)	N = 165 (%)
C2+/TT/C1+	145 (50.88)	80 (50.96)	46 (48.42)	86 (52.12)
C2+/CT/C1+	108 (37.89)	59 (37.58)	37 (38.95)	63 (38.18)
C2+/CC/C1+	32 (11.23)	18 (11.46)	12 (12.63)	16 (9.70)
	N = 210 (%)	N = 121 (%)	N = 71 (%)	N = 128 (%)
C2+/TT/C2+	106 (50.48)	60 (49.59)	34 (47.89)	67 (52.34)
C2+/CT/C2+	83 (39.52)	49 (40.50)	28 (39.44)	50 (39.06)
C2+/CC/C2+	21 (10.00)	12 (9.92)	9 (12.68)	11 (8.59)
HLA-C/ERAP1 rs2287987/HLA-C	N = 342 (%)	N = 193 (%)	N = 112 (%)	N = 231 (%)
C1+/TT/C1+	218 (63.74)	120 (62.18)	76 (67.86)	142 (61.47)
C1+/CT/C1+	103 (30.12)	57 (29.53)	31 (27.68)	81 (35.06)
C1+/CC/C1+	21 (6.14)	**16 (8.29)**[f]	5 (4.46)	8 (3.46)
	N = 260 (%)	N = 141 (%)	N = 91 (%)	N = 177 (%)
C1+/TT/C2+	171 (65.77)	90 (63.83)	65 (71.43)	111 (62.71)
C1+/CT/C2+	72 (27.69)	41 (29.08)	21 (23.08)	61 (34.46)
C1+/CC/C2+	17 (6.54)	10 (7.09)	5 (5.49)	5 (2.82)
	N = 284 (%)	N = 157 (%)	N = 94 (%)	N = 165 (%)
C2+/TT/C1+	177 (62.32)	102 (64.97)	58 (61.70)	110 (66.67)
C2+/CT/C1+	91 (32.04)	44 (28.03)	32 (34.04)	49 (29.70)
C2+/CC/C1+	16 (5.63)	11 (7.01)	4 (4.26)	6 (3.64)
	N = 210 (%)	N = 121 (%)	N = 71 (%)	N = 128 (%)
C2+/TT/C2+	136 (64.76)	82 (67.77)	45 (63.38)	87 (67.97)
C2+/CT/C2+	63 (30.00)	34 (28.10)	22 (30.99)	37 (28.91)
C2+/CC/C2+	11 (5.24)	5 (4.13)	4 (5.63)	4 (3.12)
HLA-C/ERAP2 rs2248374/HLA-C	N = 341 (%)	N = 193 (%)	N = 111 (%)	N = 231 (%)
C1+/AA/C1+	**93 (27.27)**[g]	53 (27.46)	29 (26.13)	46 (19.91)
C1+/AG/C1+	170 (49.85)	101 (52.33)	50 (45.05)	124 (53.68)
C1+/GG/C1+	78 (22.87)	39 (20.21)	32 (28.83)	61 (26.41)
	N = 258 (%)	N = 141 (%)	N = 89 (%)	N = 177 (%)
C1+/AA/C2+	**74 (28.68)**[h]	**41 (29.08)**[i]	24 (26.97)	33 (18.64)
C1+/AG/C2+	123 (47.67)	73 (51.77)	40 (44.94)	89 (50.28)
C1+/GG/C2+	61 (23.64)	**27 (19.15)**[j]	25 (28.09)	55 (31.07)
	N = 284 (%)	N = 157 (%)	N = 94 (%)	N = 166 (%)
C2+/AA/C1+	72 (25.35)	44 (28.03)	21 (22.34)	34 (20.48)
C2+/AG/C1+	141 (49.65)	79 (50.32)	43 (45.74)	86 (51.81)

Female HLA-C/male ERAP/male HLA-C	IVF	RIF	SIVF	Fertile
C2+/GG/C1+	71 (25.00)	34 (21.66)	30 (31.91)	46 (27.71)
	N = 209 (%)	N = 121 (%)	N = 70 (%)	N = 129 (%)
C2+/AA/C2+	58 (27.75)	33 (27.27)	20 (28.57)	27 (20.93)
C2+/AG/C2+	95 (45.45)	61 (50.41)	29 (41.43)	64 (49.61)
C2+/GG/C2+	56 (26.79)	27 (22.31)	21 (30.00)	38 (29.46)
HLA-C/ERAP1 rs6861666/HLA-C	N = 341 (%)	N = 191 (%)	N = 113 (%)	N = 219 (%)
C1+/AA/C1+	289 (84.75)	167 (87.43)	94 (83.19)	183 (83.56)
C1+/AG/C1+	51 (14.96)	23 (12.04)	19 (16.81)	34 (15.53)
C1+/GG/C1+	1 (0.29)	1 (0.52)	0 (0.00)	2 (0.91)
	N = 259 (%)	N = 140 (%)	N = 91 (%)	N = 167 (%)
C1+/AA/C2+	218 (84.17)	124 (88.57)	72 (79.12)	142 (85.03)
C1+/AG/C2+	40 (15.44)	15 (10.71)[k]	19 (20.88)	23 (13.77)
C1+/GG/C2+	1 (0.39)	1 (0.71)	0 (0.00)	2 (1.20)
	N = 284 (%)	N = 156 (%)	N = 95 (%)	N = 154 (%)
C2+/AA/C1+	246 (86.62)[l]	134 (85.90)	84 (88.42)	121 (78.57)
C2+/AG/C1+	37 (13.03)	21 (13.46)	11 (11.58)	30 (19.48)
C2+/GG/C1+	1 (0.35)	1 (0.64)	0 (0.00)	3 (1.95)
	N = 210 (%)	N = 121 (%)	N = 71 (%)	N = 117 (%)
C2+/AA/C2+	182 (86.67)	106 (87.60)	62 (87.32)	95 (81.20)
C2+/AG/C2+	27 (12.86)	14 (11.57)	9 (12.68)	20 (17.09)
C2+/GG/C2+	1 (0.48)	1 (0.83)	0 (0.00)	2 (1.71)

IVF-ET – in vitro fertilization embryo transfer; RIF – recurrent implantation failure; SIVF – successful pregnancy after IVF-ET; p – probability; $p_{corr.}$ – probability after Bonferroni correction for multiple comparisons (x12 for possible *HLA-C* alleles); OR – odds ratio; 95% CI – confidence interval from two-sided Fisher's exact test; ns – not significant. Values in bold indicate significant differences.

IVF *vs.* Fertile: [a]$p/p_{corr.}$ = 0.051/ns, OR = 1.480, 95% CI (0.99-2.22); [g]$p/p_{corr.}$ = 0.047/ns, OR = 1.507, 95% CI (0.99-2.31); [h]$p/p_{corr.}$ = 0.018/ns, OR = 1.753, 95% CI (1.08-2.89); [l]$p/p_{corr.}$ = 0.041/ns, OR = 1.763, 95% CI (1.02-3.05);

RIF *vs.* Fertile: [b]$p/p_{corr.}$ = 0.042/ns, OR = 1.608, 95% CI (1.01-2.58); [d]$p/p_{corr.}$ = 0.030/ns, OR = 1.655, 95% CI (1.03-2.69); [f]$p/p_{corr.}$ = 0.036/ns, OR = 2.514, 95% CI (0.99-6.95); [i]$p/p_{corr.}$ = 0.033/ns, OR = 1.786, 95% CI (1.02-3.13); [j]$p/p_{corr.}$ = 0.020/ns, OR = 0.526, 95% CI (0.30-0.92);

RIF *vs.* SIVF: [c]$p/p_{corr.}$ = 0.011/ns, OR = 0.307, 95% CI (0.11-0.82); [e]$p/p_{corr.}$ = 0.020/ns, OR = 1.914, 95% CI (1.08-3.40); [k]$p/p_{corr.}$ = 0.038/ns, OR = 0.456, 95% CI (0.20-1.01)
